# Removing direct photocurrent artifacts in optogenetic connectivity mapping data via constrained matrix factorization

**DOI:** 10.1371/journal.pcbi.1012053

**Published:** 2024-05-06

**Authors:** Benjamin Antin, Masato Sadahiro, Marta Gajowa, Marcus A. Triplett, Hillel Adesnik, Liam Paninski

**Affiliations:** 1 Mortimer B. Zuckerman Mind Brain Behavior Institute, Grossman Center for the Statistics of Mind, and Center for Theoretical Neuroscience, Columbia University, New York, New York, United States of America; 2 Department of Molecular and Cell Biology, University of California, Berkeley, Berkeley, California, United States of America; 3 Department of Statistics, Columbia University, New York, New York, United States of America; University of Edinburgh, UNITED KINGDOM

## Abstract

Monosynaptic connectivity mapping is crucial for building circuit-level models of neural computation. Two-photon optogenetic stimulation, when combined with whole-cell recording, enables large-scale mapping of physiological circuit parameters. In this experimental setup, recorded postsynaptic currents are used to infer the presence and strength of connections. For many cell types, nearby connections are those we expect to be strongest. However, when the postsynaptic cell expresses opsin, optical excitation of nearby cells can induce direct photocurrents in the postsynaptic cell. These photocurrent artifacts contaminate synaptic currents, making it difficult or impossible to probe connectivity for nearby cells. To overcome this problem, we developed a computational tool, Photocurrent Removal with Constraints (PhoRC). Our method is based on a constrained matrix factorization model which leverages the fact that photocurrent kinetics are less variable than those of synaptic currents. We demonstrate on real and simulated data that PhoRC consistently removes photocurrents while preserving synaptic currents, despite variations in photocurrent kinetics across datasets. Our method allows the discovery of synaptic connections which would have been otherwise obscured by photocurrent artifacts, and may thus reveal a more complete picture of synaptic connectivity. PhoRC runs faster than real time and is available as open source software.

## Author summary

Mapping the presence and strength of connections between neurons is necessary for understanding how the structure of neural circuits gives rise to their function. Historically, directly measuring these connections has required difficult and time-consuming paired-patch experiments. However, recent developments in the field of two-photon optogenetics enable mapping experiments with much higher throughput. In these new experiments, a single postsynaptic neuron is recorded using an electrode, and a two-photon laser is used to optogenetically activate nearby neurons. When the laser activates a connected presynaptic neuron, the experimenter observes an electrical current on the electrode. The size of the current can then be used to determine the strength of the connection. However, this technique has a drawback: when stimulating near the postsynaptic cell, the laser can directly evoke an electrical current—called a photocurrent—even if no connection is present. This is problematic because it confounds estimates of connectivity. In this work, we developed a computational tool which removes the photocurrent artifact while preserving signals corresponding to true neuronal connections. We expect that this tool will serve as a resource for experimentalists, enabling a more complete picture of synaptic connectivity.

## Introduction

Monosynaptic connectivity mapping provides essential information for constraining models of neural circuits. In visual cortex, for instance, knowledge of spatial connectivity statistics between cells offers important constraints when designing circuit models of perception [1, 2]. Two-photon optogenetics, combined with whole-cell patch clamp electrophysiology, allows the mapping of monosynaptic connectivity at a throughput not attainable with traditional paired patch electrophysiology [3–7]. In this approach, a postsynaptic neuron is recorded intracellularly while individual presynaptic neurons are excited by optogenetic stimulation. Recorded synaptic currents are then used to infer the presence and strength of monosynaptic connections. Recent computational work has shown that this approach can be scaled to test hundreds of potential connections onto a single postsynaptic partner at high speed, yielding a comprehensive spatial map of synaptic connectivity [7, 8].

Ideally, opsin would be densely expressed in the presynaptic population to allow thorough probing of possible connections. This can yield an “all-to-one” description of connectivity onto a single postsynaptic neuron. However, dense opsin expression makes it difficult or impossible to find and record from an opsin-negative cell, forcing experimenters to record from postsynaptic cells which express opsin. If the postsynaptic cell expresses opsin, photostimulation of nearby targets can directly excite the postsynaptic cell. This results in recording photocurrent artifacts, which contaminate synaptic currents and thus confound connectivity estimation. Since pyramidal cells are the most abundant cortical cell type and have relatively sparse connectivity [5, 9], dense opsin expression is especially critical when mapping connections between excitatory cells. Thus, the photocurrent artifact has been a frequent practical limitation, often forcing experimenters to discard data in which the artifact is present [5, 10].

Previously, practitioners adjusted the parameters of the experimental preparation to minimize the effect of this artifact. For example, when opsin expression is sparsely limited across the cell population, then it becomes possible to patch onto an opsin negative cell and avoid the issue altogether. However, sparse expression limits the number of connections which can be probed and therefore limits each experiment to acquiring a partial connectivity map. Thus in a previous study, practitioners aggregated partial map data across multiple experiments from different patched cells [5].

Advances in optogenetic tools, including soma-targeted opsins [4, 11, 12] and two-photon optics [13], have improved upon the effective spatial resolution of optogenetic stimulation. However, direct photocurrent artifacts still pose a major challenge when stimulating presynaptic neurons near the postsynaptic cell [5, 10]. Importantly, it is precisely in the region immediately surrounding a patched cell that we expect connectivity to be strongest for many cell types [5, 9]. Furthermore, the precise spatial statistics of connectivity are a key parameter in theories of circuit function [1, 2]. It is therefore critical to avoid systematic biases due to opsin contamination when estimating these circuit parameters.

Prior attempts to computationally remove photocurrents have relied on extensive manual curation [10], or assumed exact *a priori* knowledge of photocurrent kinetics [14] (though see [7] for an exception, discussed in more detail below). The ability to automatically detect and remove photocurrents while preserving underlying postsynaptic currents would enable more complete mapping of local connectivity without limiting the location or number of presynaptic targets. This would allow for the construction of more detailed and accurate circuit models of neural computation.

Towards these aims, we developed and validated a constrained matrix-factorization model which we call Photocurrent Removal with Constraints (PhoRC). Unlike prior approaches, PhoRC removes direct photostimulation artifacts and preserves recorded postsynaptic currents without prior knowledge of the photocurrent waveform. This means it can adapt seamlessly to variability between datasets, or even between different opsins. We validated PhoRC on simulated and real excitatory-to-excitatory mapping data, and found that it enabled consistent removal of photocurrent artifacts and thereby improved subsequent connectivity estimation.

## Results

### Two mapping protocols demonstrate the confounding effect of direct photocurrents

**Fig 1:**
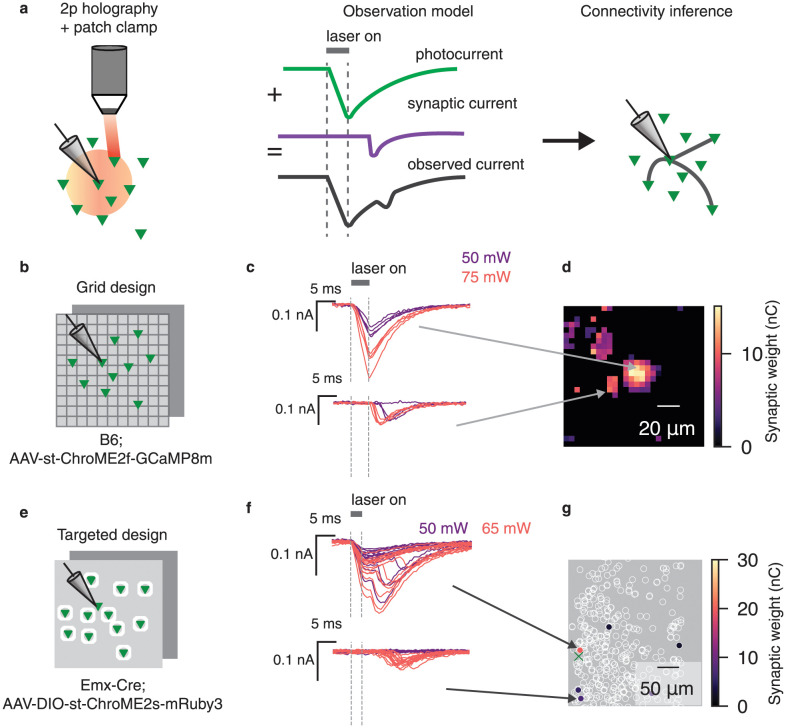
Photocurrent contamination confounds mapping of nearby synaptic connections. **a**, Synaptic connectivity mapping proceeds by patching a single downstream cell and targeting surrounding cells with two-photon excitation. When the downstream cell expresses opsin, the observed currents contain a mix of synaptic current and direct photocurrent. Left panel: cartoon of experimental setup; red circle represents radius in which direct photocurrent will be evoked. Middle panel: photocurrent artifacts are added to any observed synaptic current signals. Right panel: such artifacts subsequently corrupt connectivity inference. **b**, Schematic of grid experimental design. Stimulation laser was fired randomly in a grid pattern to excite cells expressing ChroME2f. **c**, Representative traces from a proximal (top) and distal (bottom) stimulus location in a grid experiment. Without accounting for the photocurrent artifact, both locations are naively categorized as being connected to the patched cell. The top traces represent putative photocurrent-only responses: excitatory currents which begin concurrently with laser onset, with amplitude modulated by laser power. The bottom traces represent prototypical PSC responses. Note the steeper onset profile, which occurs a few milliseconds after stimulation. PSC response latency is modulated by laser power, but amplitude is not. Purple and red traces denote 30 and 50 mW stimulation, respectively. **d**, Inferred connectivity map for an example grid experiment. The cluster of strong inferred weights in the center is likely artifactual. *Caption continued on next page.* **e**, In the targeted design, excitatory cells expressed ChroME2s fused to mRuby3, allowing us to detect and segment presynaptic target cells. **f**, Representative traces from a proximal (top) and distal (bottom) target cell in a targeted experiment. The top traces contain a putative mixture of photocurrent and synaptic current. Bottom traces represent putative PSC-only response. **g**, Inferred connectivity from a representative targeted experiment. Green cross denotes location of the postsynaptic cell.

We consider synaptic connectivity mapping experiments in which currents are recorded from an opsin-expressing postsynaptic cell, and presynaptic targets are optogenetically stimulated with a laser. In this setup, stimulation evokes direct photocurrents in the patched cell whenever the laser is used to stimulate targets that are close to the patched cell (Fig 1a, left). Direct photocurrent can be distinguished from synaptic current by its kinetics: immediate onset when stimulation begins, followed by roughly exponential decay when stimulation ends [12, 15–17]. Laser power is expected to modulate the amplitude of photocurrent response, with little impact on latency or overall kinetics [3, 15]. By contrast, typical PSCs have longer and more variable latencies compared to photocurrents, with different rise times (Fig 1a).

When the postsynaptic cell is directly photoactivated, incoming synaptic currents will be added to the induced photocurrent, creating a corrupted measurement, and subsequently biasing connectivity inference. Thus for each location stimulated, there are several possible scenarios: we may evoke pure photocurrent, pure synaptic current, or a mix of the two (Fig 1a). An effective tool for photocurrent subtraction would address all three of these cases. We therefore asked whether we could design a computational tool capable of subtracting photocurrents while preserving synaptic currents, even when they are mixed together.

We employed two mapping protocols in order to systematically study this question (Fig 1). In both sets of experiments we mapped connections between pyramidal cells by combining the scanless computer-generated holographic optogenetic system named 3D-SHOT [13] with voltage-clamp electrophysiology, and using the soma-targeted ChroME family of opsins [17]. Following similar work, we titrated laser power to almost always evoke zero or one action potentials following brief (3–5ms) pulses of stimulation [3, 5, 7]. At each stimulation, the response was taken to be the total charge transfer during a short window following stimulation (see In vitro whole-cell electrophysiology).

In the first protocol, which we term the grid-based approach, we pan-neuronally expressed soma-targeted ChroME2f, and recorded intracellular currents from an opsin-positive cell. We then stimulated points at random in a three-dimensional grid surrounding the patched cell (Fig 1b). The grid was approximately 170 *μ*m per side, and included five axial planes spaced 25 *μ*m apart (see Whole-cell grid-based synaptic connectivity mapping). Using the responses following each stimulation, we inferred connectivity using the CAVIaR software package [7]. In brief, CAVIaR uses a neural-network based denoiser, combined with a statistical model, to infer an effective synaptic weight for each presynaptic target. This yielded a spatial map in which each pixel was assigned an effective synaptic weight (Fig 1d).

We consistently found a region near the patched cell in which photocurrents dominated the observed signal (Fig 1c and 1d). The size and shape of this region varied significantly between datasets, ranging from 25–70 *μ*m in diameter (S1–S5 Figs). This led us to infer connections near the patched cell which were actually due to photocurrent artifact. By contrast, connections inferred far from the patched cell displayed typical profiles of excitatory postsynaptic currents (PSCs) (Fig 1c).

The grid-based approach allowed us to carefully characterize how photocurrent activation changes with distance and laser power, and was crucial in developing our photocurrent subtraction method. However, one possible objection to our results thus far might be that the grid-based approach includes presynaptic targets which are very close to the postsynaptic cell, and that photocurrent artifacts would have been reduced or eliminated if presynaptic cells were targeted individually, rather than stimulating blindly at a set of predetermined grid points. We therefore further investigated the impact of photocurrent artifacts using a second experimental protocol, which we term the targeted protocol.

In this protocol, we expressed a slower opsin (ChroME2s) fused to the fluorescent reporter mRuby3 in excitatory neurons. At the beginning of the experiment, two-photon imaging of mRuby3 was used to detect and segment opsin-expressing cells for subsequent stimulation (Fig 1e). As in the prior set of experiments, we observed substantial direct photocurrent when mapping cells adjacent to the patched cell, which subsequently corrupted estimates of connectivity. As in our grid experiments, inferred connections close to the patched cell frequently contained either pure photocurrent, or a mix of synaptic and photocurrent (Fig 1f and 1g), and further motivated the development of an automated photocurrent removal tool.

### A constrained low-rank model separates photocurrents from synaptic currents

**Fig 2:**
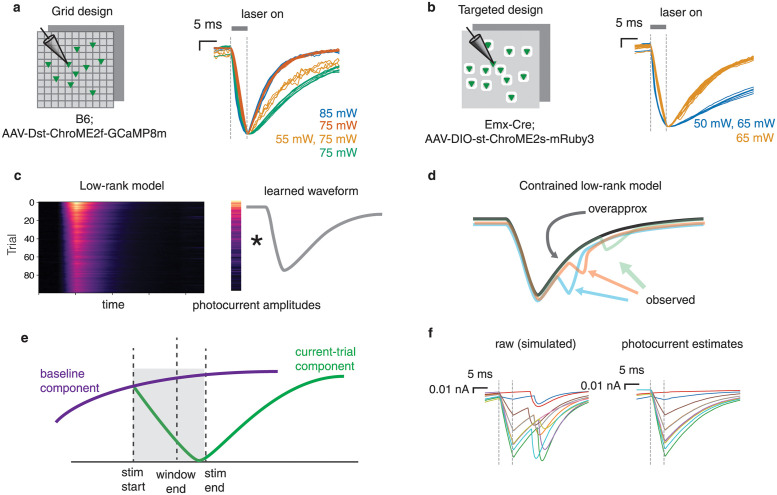
A constrained low-rank model separates synaptic currents from photocurrents. **a**, Scaled photocurrent traces from the grid experiments with ChroME2f. Colors correspond to different datasets; stimulation power used is shown on the right. Even with the same opsin, there is substantial cell-to-cell variability in the photocurrent waveform. **b**, Same as **a**, but for targeted experiments using ChroME2s. **c**, Schematic of the low-rank approach. The matrix of PSC traces (left) can be approximated as a single waveform scaled by a vector of amplitudes. **d**, The overapproximation constraint stops the estimated waveform from picking up PSCs. **e**, PhoRC estimates a baseline component which accounts for photocurrents from the prior trial. **f**, Simulated example showing the effect of the overapproximation constraint. Left: simulated traces containing a mixture of photocurrents and PSCs. Right: Photocurrent estimates for the traces shown on the left. The overapproximation constraint allows PhoRC to extract the photocurrent waveform while ignoring PSCs.

We collected four datasets using the targeted approach and two using the grid approach. For each dataset, we rescaled and plotted the ten largest traces, as determined by signal magnitude during stimulation. We found that even when using the same opsin and stimulating at similar laser powers, there was significant variability in the photocurrent waveform across datasets, possibly due to differing experimental parameters such as access resistance or slice temperature (Fig 2a and 2b). Although practioners can discard cells with high access resistance, even within the range of recordings which are normally acceptable, there can be substantial variation in access resistance, leading to variations in photocurrent and synaptic current kinetics. This variability poses a challenge to previous automated photocurrent subtraction approaches, which assume prior knowledge of the photocurrent waveform [14]. We therefore sought to develop a method which would automatically learn the photocurrent waveform from data. As we show below, pursuing such an approach has the added benefit of automatically adapting to the kinetics of different opsins.

Within a given dataset, kinetics were largely conserved across trials (Fig 2a and 2b with some exceptions, discussed below). This motivated us to consider a low-rank model in which a learned temporal waveform is scaled at each stimulation (Fig 2c). Given *N* recorded traces, each with *T* timesteps, we can express our model as

$$\begin{array}{c} Y\approx uv^{⊤}+X. \end{array}$$ (1)

Here, *Y* is an *N* × *T* matrix with recorded traces along its rows, *X* is a matrix capturing evoked PSCs at each trial, and the rank-one term *uv*^⊤^ contains our model of the photocurrent artifact, where *u* is a *N*-length vector containing the scalings, and *v* is a *T*-length vector containing the photocurrent waveform. The model described by Eq 1 is similar to low-rank methods which have been applied to the removal of electrical artifacts from extracellular array recordings [18, 19]. Unlike prior approaches which have relied on fixed templates or manual curation [10, 14] we propose to automatically learn the photocurrent waveform *v* and scalings *u* from the data itself.

The key challenge in photocurrent subtraction is to separate the photocurrent component *uv*^⊤^ from the PSC component *X*. To this end we introduce two key innovations. First, we use a short window of time during stimulation to learn the photocurrent scalings *u*. We term this section of data the *photocurrent integration window*, and in all experiments we set this window to match the time during which the laser was on. We found that this improved the model’s capacity to separate photocurrent from PSCs, since photocurrents are typically much larger than PSCs while the laser is on.

Second, we introduce an “overapproximation” constraint which requires our photocurrent estimate to lie above the recorded trace (Fig 2d, see Constrained stepwise model for more detail). This constraint leverages the fact that, compared to photocurrents, synaptic currents are much more variable. Whereas photocurrents only involve the gating of a single molecule (the opsin), synaptic currents depend on a complex array of presynaptic events—principally, the upstream spike and subsequent vesicle release—each with substantial stochasticity. Since photocurrents and PSCs are summed (Fig 1a), the photocurrent waveform present on each trial is an overapproximation of the observed current (Fig 2d). Thus, the overapproximation constraint serves to separate signal from artifact, making it possible to estimate *u* and *v* from the data despite the present of PSCs. Such constraints have been explored in the machine learning literature for robust fitting of low-rank models [20, 21], and have previously been applied when modeling barcode fluorescence data [22]. Our approach is similar to the asymmetric loss function used when processing calcium imaging data in refs. [23, 24].

Prior work on optogenetic circuit mapping has used relatively long inter-stimulus intervals (ISIs), typically around 100 milliseconds, in order to allow conductances in the postsynaptic cell to return to baseline [3–5]. However, ref. [7] demonstrated that it is possible to use much shorter ISIs and subsequently demix the overlapping signals using a neural network. In our experiments, we leveraged shorter ISIs (30–33 ms) for accelerated mapping, but found that effects from the prior or subsequent trial could confound photocurrent estimates. This was more common when using ChroME2S due to its slower decay time compared to ChroME2f. To account for this effect, we added a second low-rank component—which we called the baseline component—to capture these prior trial effects, shown schematically in Fig 2e. In order to stop the baseline component from capturing photocurrents from the current trial, we add a constraint that requires the baseline waveform to decay exponentially. This constraint can be enforced efficiently using the optimization algorithm described in ref. [25]; see Solving the NMU subproblems for details.

We found two additional modifications to the low-rank model which improved performance on real data. First, we observed small variability in photocurrent kinetics at different laser powers (S7 Fig). To ensure that this would not impact our photocurrent subtraction, we ran the low-rank approximation on batches of traces which had similar magnitude. This batching strategy had the added practical benefit of making our algorithm faster to deploy on large experiments. In practice, fixing the batch size to several hundred traces works well, and we found that performance was similar across a wide range of batch sizes (S7–S9 Figs). Choosing very small batch sizes (e.g 10–20) can result in subtraction of PSCs, but we found that all batch sizes which included 200 or more traces performed well.

Finally, we sometimes found qualitatively that a rank-two model was a better fit for the data than the rank-one model above—i.e., photocurrents are close to but not exactly scaled copies of each other (see Materials and methods). In practice, it is straightforward to select the rank of the model by subjective evaluation; we never found the need to use more than two components.

We found that the low-rank model was computationally efficient, removing photocurrents from a 25 minute experiment in under four minutes when running on a laptop. This faster than real time performance creates the potential for future online applications (see Discussion). We refer to the entire procedure for removing photocurrents as Photocurrent Removal with Constraints (PhoRC).

### PhoRC captures photocurrent artifacts across protocols and datasets

**Fig 3:**
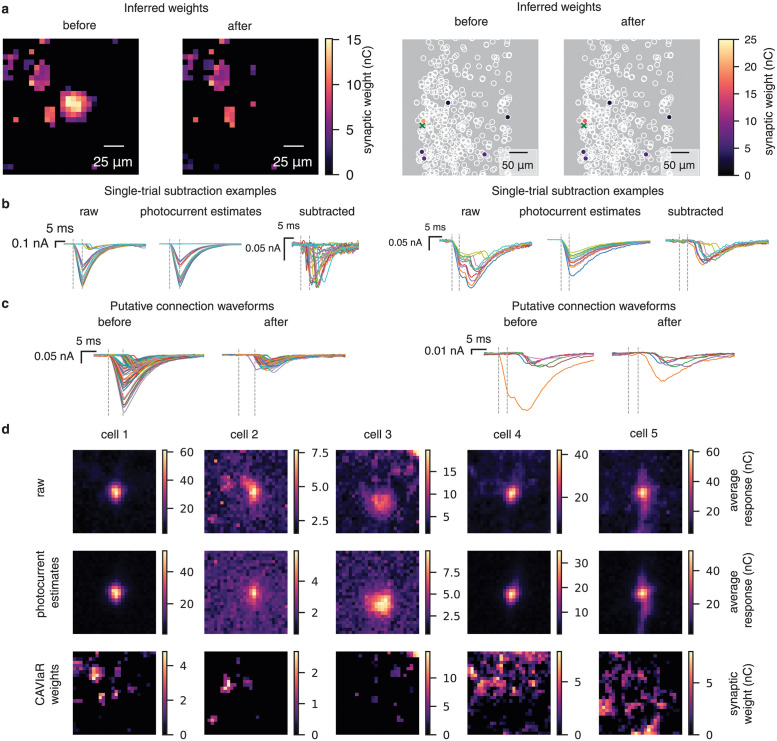
PhoRC captures photocurrent artifacts across protocols and datasets. **a**, Left: Inferred weights for the grid design, before and after subtraction. Weights are shown for a single Z plane. Right: Inferred weights before and after subtraction for a targeted experiment. Weights are shown for the entire Z stack. Green cross marks location of the postsynaptic cell. Applying PhoRC reduced the inferred synaptic strength of the connection nearest the postsynaptic cell, which was contaminated by photocurrent. **b**, Traces selected by taking 10 trials with the largest estimated synaptic current components, and ten trials with the largest estimated photocurrent components. Left panels: Observed currents, photocurrent estimates, and subtracted traces for a ChroME2f experiment. Right panels: same as in left panels but for a ChroME2s experiment. PhoRC successfully infers the photocurrent waveform in both cases, despite the use of different opsins. Estimates ignore PSCs which have higher latency than photocurrents. **c**, Left panels: average evoked waveforms for putative connections before and after subtraction for a ChroME2f experiment. Right panels: same as left, but for the ChroME2s experiment. In both cases, connection waveforms before subtraction display signs of photocurrent contamination, whereas waveforms obtained after subtraction have PSC-like profiles. **d**, PhoRC performance across grid datasets. Each column shows a different dataset. Responses are averaged across planes. From top to bottom: raw maps, photocurrent estimates, and inferred weights from the CAVIaR pipeline.

We first sought to qualitatively evaluate the estimates from PhoRC and ensure they successfully captured our intuition about which signals should be captured and which should be ignored. Using the same targeted and grid datasets shown in Fig 1, we used PhoRC to subtract photocurrents and subsequently estimated connectivity using the CAVIaR pipeline [7]. As expected, we found that PhoRC resulted in pruned connections (Fig 3a, left panels) and reduced connection strengths (Fig 3a, right panels) for targets with photocurrent contamination. For the large-scale experiments we performed, qualitative evaluation can be difficult as it is impractical to manually inspect the thousands of traces collected during a single experiment. We therefore found it useful to assess performance on two categories of traces: those with the largest putative photocurrent response, and those with the largest putative synaptic current response (see Selecting representative traces). In examining these representative traces, we found that PhoRC inferred the photocurrent waveforms while ignoring PSCs for both the grid and targeted protocols (Fig 3b). This was especially evident for traces which showed a putative mixture of photocurrents and PSCs (Fig 3b, right two panels).

Following connectivity inference, we averaged all evoked responses at the highest laser power for the targets labeled as connected. This yields a putative connection waveform which describes the average evoked current when a given target is successfully stimulated (see Connectivity inference and estimation of putative connection waveforms). With no subtraction, many of these putative connections displayed waveforms typical of photocurrent, whereas after the subtraction step, these waveforms displayed profiles typical of PSCs (Fig 3c). In some cases, the uncorrected waveforms were indicative of combined PSCs and photocurrents (Fig 3c, right two panels). In these cases, the subtracted waveforms maintained their putative PSC components while the photocurrent components were removed. The subtracted waveforms were also smaller in magnitude, reflecting the fact that, in our experiments, evoked photocurrent artifacts were often much larger than typical PSCs (0.3—0.8 nA).

For five datasets collected using the grid mapping protocol, we used PhoRC to subtract photocurrents and subsequently inferred connectivity using CAVIaR (Fig 3). Each dataset was dominated by a prominent peak of recorded current near the center of the grid, the putative location of the postsynaptic cell (Fig 3d, top). By averaging the the inferred photocurrent response at each pixel, we obtained spatial maps of evoked photocurrents (Fig 3d, middle). The scale of this photocurrent response varied signficantly between datasets, likely due to variation of opsin expression in the postsynaptic cell. After subtracting the inferred photocurrents, we recovered plausible connectivity maps which were previously obscured by the photocurrent artifact (Fig 3d, bottom). S1–S5 Figs show representative traces and the corresponding subtraction results for each of the five datasets displayed in Fig 3d. S6 Fig shows additional results for an experiment in which the postsynaptic cell is a putative parvalbumin-expressing (PV) neuron, recognizable by very quickly decaying PSCs. By examining the recorded current traces and photocurrent estimates, we found that PhoRC was able to adapt to the subtle kinetics differences we observed between datasets.

### Validating PhoRC pharmacologically and via simulated data

#### Validating PhoRC on real datasets using synaptic blockers

**Fig 4:**
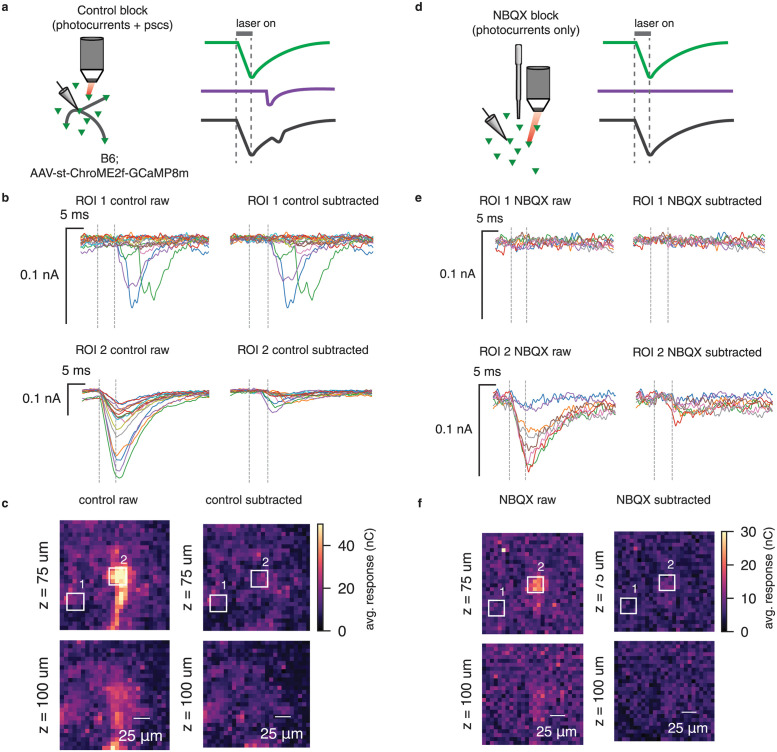
Validating photocurrent subtraction with synaptic transmission block. **a**, We used the grid design to map synaptic connections in a two part experiment. During the control block, we recorded photocurrents along with synaptic currents as in prior experiments. **b**, Raw and subtracted traces from two ROIs during the control block. ROI 1 is far from the putative location of the postsynaptic cell, while ROI 2 is nearby. **c**, Raw and subtracted grid maps from the control block. The colorbar for the “control raw” map has been truncated to improve legibility. ROIs correspond to the traces shown in **b**. **d**, After bath application of NBQX, we repeated the mapping experiment on the same patched cell, recording isolated photocurrents. **e**, Raw and subtracted traces from the NBQX block for the same two ROIs as in **b,c**. As expected, nearly all currents are removed from ROI 2. **f**, Raw and subtracted grid maps from the NBQX block. As expected, the subtracted maps during the NBQX block appear blank. ROIs correspond to the traces shown in **e**.

A critical difficulty in developing any tool for photocurrent subtraction is the lack of ground-truth data. We therefore sought to further validate PhoRC through pharamcological blockade of synaptic signaling. We first patched an opsin positive cell and mapped incoming connections using the grid protocol (Fig 4a). As expected, recorded traces contained a mix of synaptic currents and photocurrents during this control block (Fig 4b).

Following the control block, we bath applied the AMPA receptor antagonist NBQX to block synaptic transmission (NBQX block, Fig 4d), and observed that the PSCs visible during the control block were removed. If our photocurrent estimates are accurate, we would expect that PhoRC should remove all currents present during the NBQX block. Indeed, we found that nearly all currents were subtracted, resulting in a blank map devoid of photocurrents and synaptic currents (Fig 4e and 4f).

#### Validating PhoRC using simulated mapping experiments

**Fig 5:**
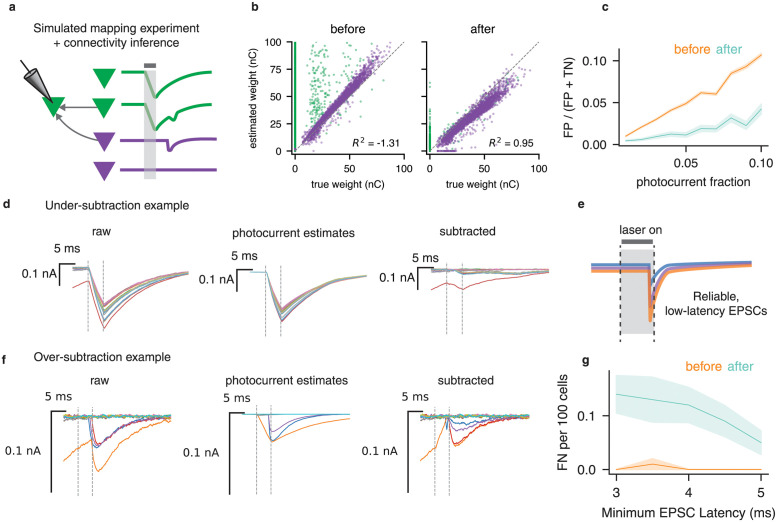
Validating photocurrent subtraction with simulated mapping experiments. **a**, We simulated complete circuit mapping experiments in which stimulating some cells (green) evoked direct photocurrent. We then used a connectivity inference pipeline to assess how photocurrent subtraction affected connectivity inference. **b**, Scatterplots of inferred vs. true weights before (left panel) and after (center panel) photocurrent subtraction. Vertical axis shows estimated weight, horizontal axis shows true weight. After subtraction, the photocurrent-inducing cells (green) are pushed closer to the identity line, while the non-photocurrent-inducing connections (purple) are largely unaffected. **c**, False positive rate as a function of the number of cells which induce direct photocurrents before (orange) and after (blue) subtraction. **d**, Traces from an example false positive in which residual current after subtraction caused us to falsely infer a connection. This typically occured due to residual photocurrent from prior trials. **e**, Schematic of low-latency PSCs, which can appear as scaled copies of one another when they are driven reliably. **f**, Example of over-subtraction that can occur in rare instances of reliable low-latency PSCs. **g**, We swept the minimum PSC latency used in a simulated experiment, and found that false negatives caused by PhoRC were most prevalent at very low latencies. However, even in this case they occurred rarely.

We next sought to understand how PhoRC affects connectivity inference. To do so, we used simulated datasets in which ground truth responses and connectivity were known. We simulated mapping experiments using a population of 100 upstream targets with ten percent connection probability. A fraction of targets, chosen at random, evoked direct photocurrent when stimulated (Fig 5a). Photocurrent waveforms were simulated using the model from ref. [16], with parameters chosen at random to capture a wide variety of photocurrent kinetics. Our simulated mapping experiments include a great deal of biological detail not directly present in the photocurrent removal model, including power-dependent PSC latency and spontaneous PSCs.

We swept the number of photocurrent inducing cells (“photocurrent fraction”) from one to ten percent and subsequently inferred connectivity. For each value of the photocurrent fraction, we used 10 random realizations of a simulated mapping experiment, in which each realization had random assignments of connectivity and photocurrent kinetics (see Validation with simulated connnectivity mapping experiments). We found significant improvement in connectivity estimates, with the coefficient of determination (*R*^2^) between true and estimated synaptic weights increasing from -1.31 to 0.95 (Fig 5b and 5d). The negative *R*^2^ in the absence of PhoRC occured due to the large number of false positives.

Applying PhoRC resulted in signficantly fewer false positive connections while preserving nearly all true connections (Fig 5b and 5d). When 10 out of 100 presynaptic targets induced a photocurrent response, the mean number of false positives was reduced from 9.62 ± 1.15 to 3.8 ± 3.75 (mean ± SD). In examining these false positive cases, we found that they were often caused by residual photocurrents from prior trials, which make it more difficult to estimate the photocurrent in the present trial. An example of this effect is shown in Fig 5e. Although our model contains a term which accounts for prior trial effects, cases in which the decay of the opsin is very slow compared to the inter-stimulus interval still present a challenge. In many cases where PhoRC did not manage to completely remove spurious connections, their inferred synaptic weight was greatly reduced (Fig 5b).

Our simulations also demonstrated that in rare cases, PhoRC can partially subtract PSCs. In our experience, this “over-subtraction” phenomenon tends to occur when two unlikely events coincide: first, all traces within a batch are free of photocurrent contamination, and second, a batch contains very low-latency PSCs which are initiated while the laser is on. When PSCs are triggered very reliably with low-latency, they can appear as scaled copies of one another, and be erroneously subtracted (Fig 5e). A representative example of this effect is shown in Fig 5f.

Fig 5g shows how the number of missed connections changes based on the minimum PSC latency used in our simulations. The effect is exceedingly rare, occurring in 0.14 ± 0.34 cells in a 100 cell population when we set the minimum PSC latency to 3 ms, a realistic minimum value for excitatory connections. This translates to roughly one erroneously subtracted connection per 700 tested connections.

#### PhoRC preserves real PSCs in hybrid simulated-plus-real datasets

In the previous section, we found rare instances where low-latency PSCs could be erroneously subtracted. However, it is difficult to assess the extent to which this may be a problem in real data, since the precise distribution of PSC latencies is unkown. To further validate PhoRC, we therefore turned to “hybrid” datasets in which simulated photocurrents were added directly to recorded PSCs. The hybrid dataset approach presents a more realistic challenge than purely synthetic data for two reasons. First, the distribution of PSC latencies more closely matches what we expect in real experiments. Second, the ground-truth recordings contain electrical noise which may not have been fully captured by our simulations. Such an approach to validation has become common practice in the spike-sorting literature, where ground-truth datasets are similarly difficult to obtain [26, 27].

**Fig 6:**
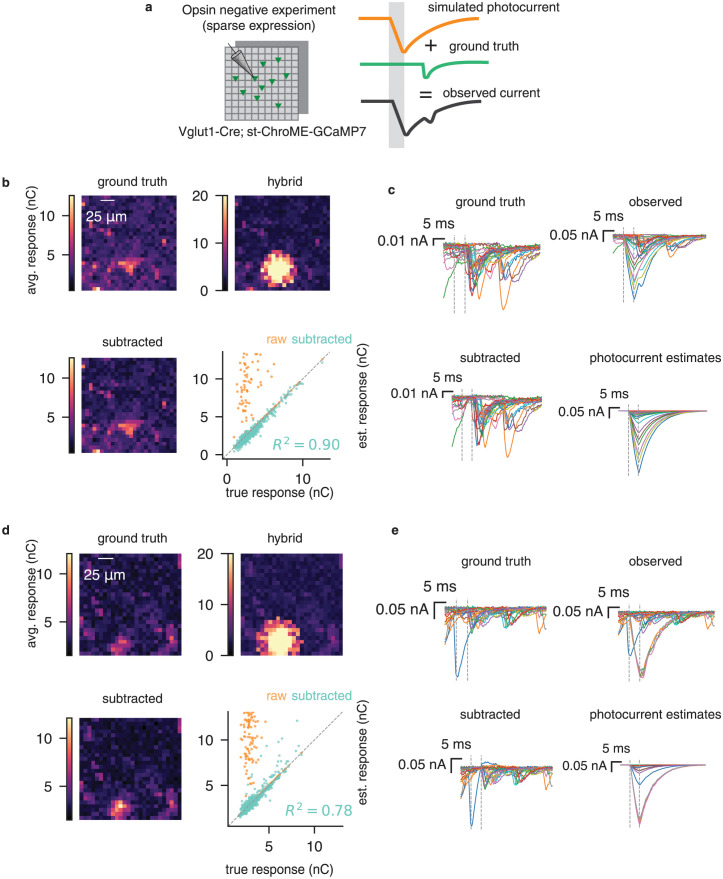
Validating photocurrent subtraction via simulated-plus-real “hybrid” datasets. **a**, Using a sparsely expressing preparation, we mapped synaptic connectivity using the grid design. We then added simulated photocurrents to these recorded PSCs in order to validate the low-rank model. **b** Ground truth PSC map, observed map containing real PSCs and simulated photocurrents, and map after subtraction. Bottom right shows the true vs. estimated responses, before and after applying PhoRC. **c**, Example traces corresponding to the maps shown in **c**. Subtracted PSCs closely mirror ground-truth. **d,e** Same as **b,c** for a second hybrid dataset.

For this set of experiments, we used the sparsely-expressing Ai203 line [28], and performed grid mapping while recording from opsin-negative cells. We then added synthetic photocurrents to these datasets, and tested our ability to recover ground-truth PSCs (Fig 6a).

Two example hybrid datasets are shown in Fig 6b–6e. We simulated photocurrent contamination by adding spatially-varying photocurrent responses, in which the mean evoked photocurrent decayed with distance from the patched cell. In order to make the experiment more challenging, we deliberately placed the peak of photocurrent contamination on top of an existing connection. After applying PhoRC, we recovered the spatial structure of the ground-truth connectivity maps (Fig 6b and 6d). If the over-subtraction problem described in the preceding section were common, we would expect that some estimated responses would be far below those of the true responses. Instead, our recovered synaptic responses closely matched the ground-truth responses on each dataset, achieving *R*^2^ values of 0.9 and 0.78 on the two datasets, respectively (Fig 6b and 6d).

### PhoRC is compatible with accelerated compressed sensing experiments

Until this point, we have considered mapping experiments in which a single upstream target is probed at a time [4, 5, 29]. However, a growing body of evidence suggests that mapping can proceed faster if a random ensemble of upstream cells is stimulated simultaneously, and connectivity is reconstructed using computational demixing approaches such as compressed sensing [3, 7, 8, 30, 31]. We refer to this experimental setup as ensemble stimulation.

**Fig 7:**
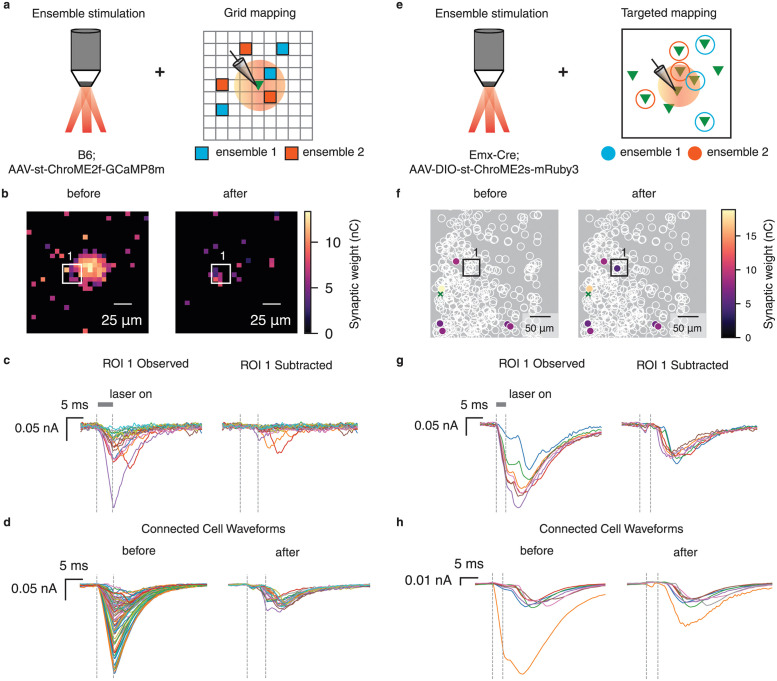
PhoRC allows ensemble mapping in the presence of photocurrent artifacts. **a**, Cartoon depicting the photocurrent contamination problem with ensemble grid stimulation. Red circle denotes region where stimulation will evoke direct photocurrent. If one grid point in an ensemble evokes photocurrent, the entire measurement will be corrupted. **b**, Inferred grid connections before and after photocurrent subtraction. ROI denotes location of a putative connection. **c**, Observed and subtracted traces from the ROI selected in **b**. Traces from this ROI contain direct photocurrents whenever a different target in the ensemble evokes direct photocurrent. **d**, Inferred waveforms for each putative connection before and after subtraction. **e-h**, Same as **a-d** but for the targeted mapping design. **e**, Cartoon depicting the photocurrent contamination problem with ensemble targeted stimulation. As in **a**, red circle denotes region where stimulation evokes direct photocurrent. **f**, Schematic of inferred connectivity before and after subtracting photocurrents, Z projection. ROI corresponds to a region far from the postsynaptic cell (green cross), but photocurrents are still present due to the use of ensemble mapping. **g**, Observed and subtracted currents from the ROI shown in **f**. **h**, Inferred waveforms for each putative connection before and after subtraction.

In the case of ensemble stimulation, photocurrent contamination has even larger effects on synaptic mapping—if even one stimulated target per ensemble induces direct photocurrent, the entire measurement will be corrupted (Fig 7a and 7e). Thus even cells which are far from the patched cell may have corrupted connectivity estimates. We therefore asked whether PhoRC can still remove photocurrents in the case of compressed sensing experiments.

For the postsynaptic cells shown in Figs 1 and 3, we additionally performed accelerated mapping with both the grid and targeted protocols in which ten targets were simultaneously stimulated through holography. Qualitatively, photocurrents induced by 10-target mapping closely resemble those induced during single-target mapping (Fig 7c and 7g, observed traces). As in the single-target mapping case, we observed relatively consistent photocurrent kinetics within a given dataset, allowing PhoRC to effectively extract the photocurrent waveform. For the grid dataset shown in Fig 7, we additionally show a comparison of synaptic weights obtained via single-target and ensemble mapping in S1 Fig.

We used the pipeline from ref. [7] to reconstruct connectivity both before and after applying PhoRC to subtract photocurrents. As before, photocurrent subtraction pruned many inferred connections near the patched cell (Fig 7b, left panels). However, in this case, we also observed that PhoRC removed photocurrents on trials which included targets far from the postsynaptic cell (Fig 7f and 7g). This reflects the fact that, in compressed sensing experiments, the photocurrent artifact can alter estimated connectivity for all presynaptic targets, not just those nearby.

We also observed that application of PhoRC to compressed sensing experiments can result in addition of connections (Fig 7b and 7f). This is because compressed sensing algorithms perform an attribution step, in which observed PSCs are attributed to a specific presynaptic cell. The photocurrent artifact interferes with this attribution step, and thus removing photocurrent can both increase and decrease inferred synaptic weights.

As in prior experiments, we found that PhoRC effectively suppressed photocurrents while preserving PSCs, and adapted accurately to the different photocurrent kinetics of the opsins used in the grid vs. targeted experiments (Fig 7c and 7g). Before applying PhoRC, many inferred connections displayed average evoked waveforms resembling photocurrents, whereas after applying PhoRC, the average waveforms matched the typical profile of PSCs (Fig 7d and 7h).

## Discussion

We developed a matrix-factorization based method called Photocurrent Removal with Constraints (PhoRC), which subtracts photocurrent artifacts while preserving synaptic current signals. In our experiments, we used soma-targeted expression of ChroME2s and ChroME2f, which reduces expression in neurites and thereby limits the region proximal to a cell of interest in which off-target stimulation evokes direct photocurrent [11, 12, 17]. Nonetheless, we observed confounding photocurrent artifacts when using two-photon holographic optogenetics to map synaptic connections near the postsynaptic cell. After applying PhoRC, we were able to map these connections despite confounding photocurrent effects. PhoRC was able to infer the photocurrent waveform for two opsins with different kinetics, and subsequently improved the accuracy of two approaches for synaptic mapping (grid-based and target-based) and across both simulated and hybrid datasets. Via our synaptic blocker validation experiment, we verified that PhoRC is able to achieve near-complete subtraction of photocurrents.

Finally, we found PhoRC to be compatible with compressed-sensing based mapping experiments in which ensembles of candidate presynaptic cells are excited simultaneously. Compatibility with compressed sensing is crucial given recent evidence that such techniques can accelerate mapping by nearly an order of magnitude [7, 8]. PhoRC is especially applicable to compressed sensing experiments given that the probability of observing the photocurrent artifact grows with the number of targets in the ensemble. In our ensemble mapping experiments, we found that PhoRC removed confounding photocurrents even for ensembles which contained targets far from the postsynaptic cell. Thus, we predict that the combination of PhoRC with compressed sensing will allow mapping of both near and far connections onto a single neuron.

PhoRC uses a short window of time during stimulation to estimate the amount of photocurrent contamination present in each response. In all cases, we set this parameter to match the duration of photostimulation (either 3 or 5 ms) for our experiments. However, if an experiment uses stimulation durations exceeding 5 ms, practitioners may find it useful to reduce this window to around 5 ms, rather than matching it exactly to the duration of photostimulation.

As with any computational tool, some care must be taken when interpreting the outputs of PhoRC. In some cases, PhoRC can leave residual photocurrents, leading to false positive connections. Although the model includes terms which account for residual photocurrent from prior trials (see Constrained stepwise model), we found in simulation that these residual effects could present challenge cases for PhoRC. These cases are more common when using short inter-stimulus intervals and slower opsins.

There has been limited prior work on removing the photocurrent artifact. Printz *et al* manually selected traces which contained only photocurrent, and averaged these traces to extract a separate photocurrent waveform for each presynaptic target [10]. The authors also noted that they were forced to discard data in which the artifact was larger than a certain threshold. This manual strategy implicitly assumes that photocurrent amplitude is identical between successive stimulations of the same presynaptic target. By contrast, the low-rank model used here adapts seamlessly to random variation in photocurrent amplitude.

Merel and Shababo *et al* developed a Bayesian framework in which photocurrents could be removed given knowledge of the photocurrent waveform; however, this approach requires *a priori* knowledge of photocurrent kinetics, and assumes that these kinetics are independent of applied laser power [14]. Unlike these prior approaches, PhoRC learns the photocurrent waveform from the data itself and can adapt to power-dependent changes in the temporal shape of the photocurrent, allowing practitioners to use their entire dataset without time-consuming manual removal of artifacts.

One other related approach is that of [7], which used a non-linear signal processing technique called neural waveform demixing (NWD). NWD uses a neural network trained on simulated data to denoise recorded PSC traces and correct for confounded baselines. In some regimes, NWD can effectively separate PSCs from photocurrents due to the difference in latency. In our experiments, we found that NWD reduced the impact of photocurrents, but did not eliminate them. Indeed, all connectivity estimates shown above (e.g Fig 1d and 1g) used NWD as a preprocessing step before connectivity inference.

In the process of developing PhoRC, we also explored the possibility of using a neural network (as in NWD) to pool information across recorded traces and thereby extract the photocurrent waveform [32]. While this approach worked well, we found that it required costly re-training of networks for different stimulus durations. We therefore adopted the low-rank model described here, which achieved similar accuracy and adapts easily to different pulse durations without requiring any laborious neural network retraining. In certain cases, the noise-robustness of a neural network approach may be preferred, and we leave this as an avenue for future work.

Several possible extensions of PhoRC may increase its applicability. First, while we demonstrated post-hoc removal of photocurrents following the completion of an experiment, it may be useful in future applications to remove photocurrents while an experiment is in-progress. Running on a laptop, PhoRC runs faster than real time and is therefore well suited to online applications. This creates the potential for adaptive experiments in which practitioners are able to immediately identify putative connections amidst photocurrent artifacts, and target these putative connections for further study. Finally, while we have considered mapping experiments in acute slices, *in vivo* connectivity mapping might provide deeper insights into the circuit mechanisms engaged during behavior. The *in vivo* setting may require extensions of PhoRC to handle increased spontaneous activity and more frequent recording instability.

We view PhoRC as one method in an increasingly broad suite of computational tools which allow two-photon optogenetics to thoroughly characterize monosynaptic connectivity [7, 8, 30, 33]. PhoRC is compatible with methods for accelerated mapping via compressed sensing, and thus we predict it will enable experimenters to measure both short and long-range connectivity in a single experiment. Such experiments would contribute to a more complete view of neural connectivity, and could subsequently inform improved models of neural circuits.

## Materials and methods

### Experimental methods

#### Ethics statement

All surgical procedures and experiments on animals were conducted in compliance with the Animal Care and Use Committee of the University of California, Berkeley under protocol AUP-2014-10-6832-3.

#### Animals

The mice used for experiments in this study were C57BL/6 (B6; JAX stock#000664) and Emx1-Cre (JAX stock# 005628), with the latter further crossed with CD-1 (ICR; Charles River) mice. Mice were housed in cohorts of five or fewer in a reverse light:dark cycle of 12:12 hours, with experiments occurring during the dark phase. All experiments used male and female mice equally.

#### Viral expression of opsins

B6 or Emx-Cre mice were neonatally injected with adeno-associated virus (AAV)-driven vectors to pan-neuronally express Chrome2f via the synapsin promoter (AAV9.hSYN.ChroME2f-6xHIS.GCaMP8m-ST.SV40) or selectively express Chrome2s in excitatory neurons in a cre-dependent manner (AAV9.CAG.DIO.ChroME-ST.P2A.H2B-mRuby6), respectively. Both custom made viral preparations for Chrome2f and 2s were generated by the Penn Vector Core. Pups aged P3–5 were anesthetized by placing them on ice for approximately 3 minutes. Each animal was then stabilized and virus was injected using a Nanoject III nanoliter injector (Drummond Scientific Company) in 4 sites surrounding the area of V1. For each site, virus was injected with 30 nl per injection at 5 depths approximately spanning the cortical layers. After injections, the animal was placed on a heating pad for post-surgical recovery. All injected pups were returned to home cage with parent mouse and housed together until weaning age (P21).

#### 3D-SHOT holography

All in vitro electrophysiology experiments employed 3D scanless holographic optogenetics with temporal focusing (3D-SHOT), as described previously [7, 12, 17]. Based on the Movable Objective Microscope (MOM; Sutter Instrument Co.) platform, the setup is built with three combined optical paths: a 3D two-photon (2p) photostimulation path, a fast resonant-galvo raster scanning 2p imaging path, and a widefield one-photon (1p) epifluorescence/IR-transmitted imaging path, merged by a polarizing beamsplitter (PBS) before the microscope tube lens and objective. A Monaco 1035-80-60 737 (1040nm, 1MHz, 300fs, Coherent Inc.) fiber laser was used for photostimulation and a Mai Tai Ti:sapphire laser (Spectra Physics Inc.) was used for 2p calcium imaging. Temporal focusing of the photostimulation beam from the femtosecond fiber laser was achieved with a blazed holographic diffraction grating (33010FL01-520R Newport Corporation). The beam was then relayed through a rotating diffuser to randomize the phase pattern and to expand the temporally focused beam to cover the area of the high-refresh-rate spatial light modulator (SLM; HSP1920-1064-HSP8-HB, 1920 × 1152 pixels, Meadowlark Optics). Holographic phase masks were calculated using the Gerchberg-Saxton algorithm and displayed on the SLM to generate multiple temporally-focused spots in 2D or 3D positions of interest [12]. The photostimulation path was then relayed into the imaging path with a PBS placed immediately prior to the tube lens. As described in [12], to limit imaging artifacts introduced by the photostimulation laser, the photostimulation laser was synchronized to the scan phase of the resonance galvos using an Arduino Mega (Arduino), gated to be only on the edges of every line scan.

### In vitro whole-cell electrophysiology

In vitro slice recordings were performed on 300*μ*m-thick coronal slices obtained from both male and female mice aged P24–40. Slice preparation followed previously described methods [7, 13, 28]. For each mouse brain to be prepped, opsin expression was checked using a handheld laser light to visualize fluorescence of the mRuby reporter (red; for Chrome2s) or fused GCaMP8 (green; for Chrome2f). Whole-cell patch-clamp protocols were performed in with heating-controlled (33°C) standard ACSF bath solution (in mM: NaCL 119, NaHCO3 26, Glucose 20, KCl 2.5, CaCl 2.5, MgSO4 1.3, NaH2PO4 1.3). Patch pipettes (4–7 MOhm) were pulled from borosilicate glass filaments (Sutter Instrument Co.) and filled with Cesium (Cs2+)-based internal solution (in mM: CeMeSO4 135, NaCl 3, HEPES 10, EGTA 0.3, Mg-ATP 4, Na-GTP 0.3, Qx-314 1, TEA-Cl 5, 295 mOsm, pH = 7.45) also containing 50 *μ*M Alexa Fluor hydrazide 488 or 594 dye (ThermoFisher Scientific). Data was recorded at 20 kHz using 700b Multiclamp Axon Amplifier (Molecular Devices). The headstage and electrode holder (G23 Instruments) was controlled by Motorized Micromanipulator (MP285A; Sutter Instrument Co.). All data was acquired and analyzed with custom code written in Matlab using the National Instruments Data Acquisition (DAQ) Toolbox (NI). Membrane (Rm) and series (Rs) resistance were monitored through short sessions of hyperpolarizing steps before and throughout experiments to ensure quality of acquired data. For all experiments, trials with Rs exceeding 30 MOhm were excluded from data analysis. At each stimulation, the response was taken to be the total charge transfer during a 35ms window following stimulation.

#### Whole-cell grid-based synaptic connectivity mapping

Cells were patched in whole-cell configuration and voltage clamped at -70 mV. Preliminary assessment of photostimulation-evoked postsynaptic responses used widefield 1P stimulation at saturating power. To map synaptic inputs to the patched cell, the surrounding volume of 162.5 × 162.5 *μ*m in lateral (x, y) and 100 *μ*m in axial (z) dimensions was probed through holographic photostimulation of a precomputed three-dimensional grid of holographic spots. Each point (voxel) of photostimulation was spaced 6.5 × 6.5 × 25 (x, y, z) *μ*m apart, targeted randomly at 30 or 40 Hz with 5 ms laser pulses across 20 trials per laser power tested. To build the grid maps, responses at each trial were calculated as the integral of the recorded current for each trial, and theses responses were averaged for all stimulations at a given voxel.

#### Whole-cell targeted synaptic connectivity mapping

Whole-cell targeted mapping experiments were performed as previously described [7]. An opsin-negative L2/3 putative pyramidal neuron (based on cell body shape) was sealed onto with a recording pipette and the area surrounding the target cell was imaged at 40 frames at 4–5 planes spaced by 25 *μ*m. The positions of the opsin-positive presynaptic candidates were automatically identified in a specified FOV using an in-house algorithm detecting mRuby3 fluorescence in round shapes representing cell nuclei. Next, 10–20 different holograms of either single or ensemble sets of candidate presynaptic targets were computed (see Methods: 3D-SHOT Holography). During hologram computation, stable whole-cell configuration was established in the patched cell and clamped at -70mV. Presynaptic candidates were mapped individually or in 10-target ensembles and across 3–4 powers and repeated 7–15 times per condition in randomly interleaved experimental trials. A map containing 100 presynaptic candidates would contain data from 45 trials of 100 single-target holograms across 3 powers (15 trials/power condition) and 45 trials of 100 different 10-target holograms (100 presynaptic candidates split into 10 different 10-target holograms that were then arranged randomly in 10 sets) across 3 powers (15 trials/power condition), resulting in 495 total trials (45 single-target sets and 450 10-target sets).

### The PhoRC algorithm

#### Unconstrained model via SVD

To build intuition, we begin with a simplified version of the model, and then add constraints and modifications as needed. For simplicity in this section, we reverse the sign of excitatory currents, so that an upwards deflection represents inward current. To begin, assume we have *N* intracellular traces, each with *T* timesteps. We begin by thresholding these traces at zero, since any deflections below zero are due to noise. Let *Y* denote this thresholded matrix *N* × *T*, in which recorded traces are stacked along its rows.

If we plot the rows of the matrix *Y*, we see that the shape of the photocurrent waveform remains largely consistent across stimulations within a single experiment (Fig 2a, 2b and 2c). The amplitude of the artifact, however, varies significantly across stimulations, since variable amounts of opsin are excited depending on stimulus location and power. This suggests approximating *Y* using a rank-one matrix, in which a single temporal waveform *v* is scaled at each trial by a parameter *u*_*i*_. We can write this simple model as *Y* = *uv*^⊤^ + *X*, where *Y* is the matrix of observed traces, and *X* is an (unobserved) matrix containing PSCs. Our strategy will be to estimate *u* and *v* in order to ultimately recover *X*. Both *u* and *v* can be estimated by solving the following optimization problem, whose solution is obtained via the SVD of *Y*:

$$\begin{array}{cc} \underset{u,v}{\text{min}} & ∥Y-uv^{⊤}{∥}_{F}^{2}. \end{array}$$ (2)

In our experiments, we sometimes observed that photocurrents were not exactly scaled copies of each other. In these cases, we found it beneficial to approximate the photocurrent artifact as a sum of *R* temporal waveforms:

$$\begin{array}{c} Y=X+u_{1}v_{1}^{⊤}+u_{2}v_{2}^{⊤}+\ldots u_{R}v_{R}^{⊤}. \end{array}$$ (3)

Setting *R* to larger values leads to more complete photocurrent subtraction, at the cost of possible subtraction of PSCs (see Setting PhoRC hyperparameters). This model can be compactly written as *Y* = *UV* + *X* where *U* is an *N* × *R* matrix *U*, and an *V* is an *R* × *T* matrix. However, this simple approach fails because the low-rank term *UV* frequently includes PSCs in addition to photocurrents. We therefore introduced constraints which incorporate our prior knowledge about the interaction between PSCs and photocurrents.

#### Constrained stepwise model

Recall that in this section, we are working with the negative of the recorded traces, so that photocurrents and PSCs are represented as positive deflections from baseline. Since excitatory synaptic currents and photocurrents sum linearly (assuming a perfect space-clamp), the photocurrent waveform present on each trial should be strictly smaller than than the observed trace. We can incorporate this prior knowledge as an under-approximation constraint, which will stop the waveforms *V* from including PSCs. Further, we wish for the weights *U* to encode the strength of the photocurrent artifact at stimulation. If we were to learn *U* using the entire data matrix *Y*, then *U* would include information about the strengths of both photocurrents and PSCs. To avoid this, we instead optimize *U* on a short window of data around laser onset, before most upstream spikes have had time to propagate. These two modifications to the model in Eq 2 give rise to what we term a constrained stepwise model.

We proceed in two stages. First, we fit *U* on a short window of data, which we call the photocurrent integration window. Let *t*_1_ denote the beginning of this window, and *t*_2_ denote the end (see below for how these values can be selected). We learn trial-weights *U*_stim_ by solving

$$\begin{array}{ll} \underset{U_{\text{stim}},V_{\text{stim}}}{\text{minimize}} & ∥Y_{:,t_{1}:t_{2}}-U_{\text{stim}}V_{\text{stim}}{∥}_{F}^{2}\\ \text{subject}\;\text{to} & U_{\text{stim}}\geq 0,V_{\text{stim}}\geq 0,\\ \; & U_{\text{stim}}V_{\text{stim}}\leq Y_{:,t_{0}:t_{2}}. \end{array}$$ (4)

This is a nonnegative matrix underapproximation (NMU) problem. Though it is non-convex, good solutions can be reached efficiently using the alternating directions method of multipliers (ADMM, detailed below). For computational efficiency, we follow the approach used in [20, 21], in which a single rank-one component $u_{i}v_{i}^{⊤}$ is learned at a time, and then subtracted from the data matrix *Y*. Recursively, we then define $Y\leftarrow Y-u_{i}v_{i}^{⊤}$ and proceed to fit the next rank-one component.

After solving Eq 4, we have obtained values for the photocurrents weight *U*_stim_. We also obtained estimates of the photocurrent waveform(s) *V*_stim_. However, these waveform estimates only account for the short section of data during the photocurrent integration window, from *t*_1_ to *t*_2_. In step two of the PhoRC algorithm, we hold our learned weights constant, and infer waveforms for the entire trial:

$$\begin{array}{ll} \underset{V}{\text{minimize}} & ∥Y-U_{\text{stim}}V{∥}_{F}^{2}\\ \text{subject}\;\text{to} & \begin{array}{c} V\geq 0,\;U_{\text{stim}}V\leq Y. \end{array} \end{array}$$ (5)

Our estimate of the photocurrent is then *U*_stim_*V*, which we subtract away to yield the underlying PSCs. Conceptually, these two steps form the core of the PhoRC algorithm. However, we found it helpful to introduce additional constraints in some cases, which we detail below.

#### Accounting for prior trial effects and corrupted baselines

Up to this point, our model has implicitly assumed that the postsynaptic cell has returned to its baseline at stimulation onset. We found that at the short ISIs used in our experiments (30–33 ms), effects from the prior or subsequent trial could confound photocurrent estimates. This was more common when using ChroME2S due to its slower decay time compared to ChroME2f. To account for this effect, we add a second low-rank component which captures these prior trial effects. Further, we found that some recorded traces had nonzero baselines, which we accounted for using a constant baseline term. With these additional terms, our model can be written as

$$\begin{array}{c} Y=X+U_{\text{base}}V_{\text{base}}+U_{\text{stim}}V_{\text{stim}}+\beta 1^{⊤} \end{array}$$ (6)

Here, *U*_base_*V*_base_ is a low-rank term which will approximate decaying photocurrents left over from the prior trial. As before, *U*_stim_*V*_stim_ captures photocurrents from the current trial. Finally, *β* **1**^⊤^ forms a matrix with constant rows, allowing a nonzero offset for each recorded trace. Finally, we add constraints so that each term in the model behaves as desired. As before, fitting proceeds in two steps, leading to the full PhoRC algorithm described in Alg 1. In the first step, we solve the following minimization:

$$\begin{array}{ll} \underset{u_{\text{base}},v_{\text{base}}u_{\text{stim}},v_{\text{stim}},\beta }{\text{minimize}} & ∥Y_{:,t_{0}:t_{2}}-\left(U_{\text{base}}V_{\text{base}}+U_{\text{stim}}V_{\text{stim}}+\beta 1^{⊤}\right){∥}_{F}^{2}\\ \text{subject to} & U_{\text{stim}}\geq 0,V_{\text{stim}}\geq 0,U_{\text{base}}\geq 0,V_{\text{base}}\geq 0,\\ & \beta \geq 0,U_{\text{stim}}V_{\text{stim}}+U_{\text{base}}V_{\text{base}}+\beta 1^{⊤}\leq Y_{:,t_{0}:t_{2}},\\ & \begin{array}{c} V_{\text{base},i}\leq \gamma V_{\text{base},i-1}\;i=2,\ldots t_{2}. \end{array} \end{array}$$ (7)

Each constraint has a simple interpretation. The first set of constraints enforces nonnegative weights, waveforms, and offsets. The second set of constraints is the underapproximation constraint, which stops the low-rank terms from including PSCs. In our experiments, we use a rank-one model to capture previous trial effects, so *U*_base_ is a column vector and *V*_base_ is a row-vector.

In the final set of constraints above, *γ* is a hyperparameter between zero and one. Thus, this set of constraints ensures that *V*_base_ decreases exponentially over time, which forces this term to capture baseline effects from the previous trial. The hyperparameter *γ* should be chosen to bound the decay of the opsin, and we found that a value of *γ* = 0.999 worked well for the ChroME opsins used in our experiments. Such a constraint has previously been used to model fluorescence from calcium imaging data, in which an exponential decrease in fluorescence is expected following a spike [25]. As we will show below, we can therefore leverage optimization strategies originally developed in the context of calcium imaging.

To solve this problem efficiently we use a coordinate descent approach, updating each rank-one term while holding the others fixed. Empirically, we found that only a few (5–10) coordinate descent iterations were needed to obtain good estimates. Full pseudocode for this algorithm is provided in Alg 2.

The second step of our algorithm remains conceptually the same as in Constrained stepwise model. After solving Eq 7, we have obtained values for the relative weights of previous trial photocurrent (*U*_base_), present trial photocurrent (*U*_stim_), and constant baselines (*β*). In step two of the PhoRC algorithm, we hold our learned weights constant, and infer waveforms for both low-rank terms:

$$\begin{array}{ll} \underset{V_{\text{base}},V_{\text{stim}}}{\text{minimize}} & ∥Y-\left(U_{\text{base}}V_{\text{base}}+U_{\text{stim}}V_{\text{stim}}+\beta 1^{⊤}\right){∥}_{F}^{2}\\ \text{subject to} & V_{\text{stim}}\geq 0,V_{\text{base}}\geq 0,\\ & U_{\text{stim}}V_{\text{stim}}+U_{\text{base}}V_{\text{base}}+\beta 1^{⊤}\leq Y,\\ & V_{\text{base},i}\leq \gamma V_{\text{base},i-1}\;i=2,\ldots t_{2},\\ & V_{\text{stim},i}\leq \gamma V_{\text{stim},i-1}\;i=t_{3},\ldots ,T. \end{array}$$ (8)

Note that Eq 8 is nearly identical to problem Eq 7. The key difference is that in Eq 7 we use only a short section of the data matrix, and optimize over both weights and waveforms. In Eq 8 we hold weights fixed, using the full dataset to optimize only over the waveforms. The final set of constraints in Eq 8 work to prevent the photocurrent waveform from picking up effects from the *subsequent* trial. The time index *t*_3_ is a user defined hyperparamter that sets the index at which we enforce the exponential decay constraint. In practice, setting *t*_3_ to be 10 milliseconds after stim offset works well. The precise value of this parameter does not have a large impact on results. A schematic showing the role of the parameters *t*_1_, *t*_2_, *t*_3_ is given in S10 Fig.

#### Solving the NMU subproblems

The PhoRC algorithm requires solving several NMU subproblems. In the case where we are not concerned with overlapping trial effects, these problems have the following form:

$$\begin{array}{ll} \underset{U,V}{\text{minimize}} & ∥Y-UV{|}_{F}^{2}\\ \text{subject to} & \begin{array}{c} U\geq 0,\;V\geq 0,\;UV\leq X. \end{array} \end{array}$$ (9)

This problem can be solved by fitting a single rank-one component at a time using ADMM. We refer the reader to [21] for details of this approach. In order to account for prior-trial and next-trial effects, we needed to introduce the exponential decay constraints shown in Eqs 7 and 8. Updating a rank-one term in that case results in the following sub-problem:

$$\begin{array}{ll} \underset{u,v}{\text{minimize}} & ∥Y-uv^{⊤}{∥}_{F}^{2}\\ \text{subject to} & u\geq 0,\;v\geq 0,\\ & uv^{⊤}\leq Y,\\ & F\left(\gamma \right)v\leq 0, \end{array}$$ (10)

where *F*(*γ*) is a first difference matrix with hyperparameter *γ*:

$$\begin{array}{c} F=[\begin{array}{ccccc} -\gamma & 1 & \ldots & 0\\ 0 & -\gamma & 1 & \ldots & 0\\ \; & \ddots & \\ 0 & \ldots & 0 & -\gamma & 1 \end{array}]. \end{array}$$ (11)

In the case that *γ* = 1, the last constraint in problem 10 becomes a monotonic decay constraint. Our approach will be to optimize simultaneously over *u*, *v* using ADMM [34]. We begin by rewriting problem 10 using auxiliary variables:

$$\begin{array}{ll} \underset{u,v,q,R}{\text{minimize}} & ∥R{∥}_{F}^{2}\\ \text{subject to} & Y-uv^{⊤}=R,\;q=v,\\ & u\geq 0,\;v\geq 0,\;R\geq 0,\\ & uv^{⊤}\leq Y,\\ & \begin{array}{c} F\left(\gamma \right)q\leq 0. \end{array} \end{array}$$ (12)

We also introduce dual variables Λ which will be used to enforce the constraint that *Y* − *uv*^⊤^−*R* = 0 and λ, which will be used to enforce the constraint that *q* − *v* = 0. The ADMM udpate for *q* requires the following minimization:

$$\begin{array}{ll} \underset{q}{\text{minimize}} & \lambda^{⊤}q+\frac{\rho }{2}∥q-v{∥}_{2}^{2}\\ \text{subject to} & F\left(\gamma \right)q\leq 0. \end{array}$$ (13)

Noting that

$$\begin{array}{c} \lambda^{⊤}q+\frac{\rho }{2}{∥q-v∥}_{2}^{2}\propto \frac{\rho }{2}{∥q-\left(v-\frac{1}{\rho }\lambda \right)∥}_{2}^{2}, \end{array}$$ (14)

where the proportionality hides terms which are constant with respect to *q*, we can write the above problem as

$$\begin{array}{ll} \underset{q}{\text{minimize}} & ∥q-\left(v-\frac{1}{\rho }\lambda \right){∥}_{2}^{2}\\ \text{subject to} & q_{2}\leq \gamma q_{1},\;q_{3}\leq \gamma q_{2},\;\ldots \;q_{N}\leq \gamma q_{N-1}. \end{array}$$ (15)

When *γ* = 1 this problem can be solved in O(T) time by the the Pool Adjacent Violators Algorithm (PAVA) [35]. In the case of 0 < *γ* < 1, Friedrich *et al* developed an extension of the PAVA algorithm which efficiently solves this case as well [25]. The update for *q* has an appealing interpretation: it is a projection of the point $v-\frac{1}{\rho }\lambda$ onto the convex set defined by the constraints [34]. When modeling the current-trial photocurrent waveform (*V*_stim_) we are only concerned with enforcing the decay constraint on a subset of the entries. In this case, we simply apply the projection operator to a subset of the entries of *q*.

We can compute the update for *v* similarly. Let *Q* = *Y* − *R*. The update for *v* requires solving the following minimization:

$$\begin{array}{ll} \underset{v}{\text{minimize}} & -\text{Tr}\left(\Lambda^{⊤}uv^{⊤}\right)-\lambda^{⊤}v+\frac{\rho }{2}∥Q-uv^{⊤}{∥}_{F}^{2}+\frac{\rho }{2}∥q-v{∥}_{2}^{2}\\ \text{subject to} & \begin{array}{c} v\geq 0. \end{array} \end{array}$$ (16)

This problem separates along the entries of *v*, and so we can take the gradient of the objective and set it to zero, then threshold the resulting solution. This yields

$$\begin{array}{c} v_{k+1}={\left[\frac{{\left(\Lambda +\rho Q\right)}^{⊤}u+\lambda +\rho q}{\rho (∥u{∥}_{2}^{2}+1)}\right]}_{+} \end{array}$$ (17)

Updates for the other variables are unchanged from the original underapproximation problem considered in [21]. We present pseudocode for fitting a rank-one component with both exponential decay and underapproximation constraints in Alg 3. In this function, PAVADecreasing implements a projection onto the set of exponentially decreasing vectors using the OASIS algorithm from [25].

**Algorithm 1:** Photocurrent Removal with Constraints (PhoRC)

**input**: PSC traces *Y*, ADMM penalty parameter *ρ*, convergence tolerance *ϵ*, decay *γ*, rank *R*, stimulation start index *t*_1_, window end index *t*_2_, decay start index *t*_3_

**1** Set $U_{\text{base}},V_{\text{base}},U_{\text{stim}},V_{\text{stim}},\beta \leftarrow \text{NMU-Coordinate-Descent}\left(Y_{0:t_{2}},\rho ,\epsilon ,\gamma \right)$

**2** Fit decaying prior-trial term *V*_base_ with fixed weights *V*_base_ ← NMUDecreasing(*Y*, *U*_base_, *V*_base_, *γ*, update_u=False)

**3** Subtract prior-trial and baseline terms *X* ← *Y* − *U*_base_*V*_base_ − *β***1**^⊤^

**4 for** *r = 1,…,R* **do**

**5**  Fit photocurrent component *r* with fixed weights *V*_base,*r*_ ← NMUDecreasing(*Y*, *U*_base,*r*_, *V*_base,*r*_, *γ*, update_u=False, *t*_3_)

**6**  Subtract rank-one photocurrent term *Y* ← *Y* − *U*_base,*r*_, *V*_base,*r*_

**7 end**

**8 return** *U*_stim_, *V*_stim_

**Algorithm 2:** NMU-Coordinate-Descent

**input**: PSC traces up to end of integration window $Y_{0:t_{2}}$, ADMM penalty parameter *ρ*, convergence tolerance *ϵ*, decay *γ*, rank *R*, stimulation start index *t*_1_, window end index *t*_2_, iterations *K*

**1** Initialize *U*_base_, *V*_base_ on window of data before stim starts: $U_{\text{base}},V_{\text{base}}\leftarrow \text{NMUDecreasing}\left(Y_{0:t_{1}},U_{\text{base},r},V_{\text{base},r},\gamma ,\text{update\_u}=\text{True}\right)$

**2** Extend *V*_base_ to include all timesteps until window end: $V_{\text{base}}\leftarrow \text{NMUDecreasing}\left(Y_{0:t_{2}},U_{\text{base}},V_{\text{base}},\gamma ,\text{update\_u}=\text{False}\right)$

**3** Initialize photocurrent components using data during integration window $U_{\text{stim}},V_{\text{stim}}=\text{NMUDecreasing}\left(Y_{t_{1}:t_{2}}-U_{\text{base}}V_{\text{base}},U_{\text{stim}},V_{\text{stim}},\gamma ,\text{update\_u}=\text{True},t_{3}\right)$

**4** Initialize baseline offsets using pointwise minimum of residual:

**5** $\beta \leftarrow {\text{min}}_{t}{\left(Y-U_{base}V_{\text{base}}-U_{\text{stim}}V_{\text{stim}}\right)}_{t}$

**6** *β* ← max(*β*, 0)

**7** **for** *k = 1*,…,*K* **do**

**8**  Set residual matrix $\tilde{Y}=Y_{0:t_{2}}-U_{\text{stim}}V_{\text{stim}}-\beta 1^{⊤}$

**9**  update prior-trial components components using residual $U_{\text{base}}V_{\text{base}}\leftarrow \text{NMUDecreasing}\left(\tilde{Y},U_{\text{base}},V_{\text{base}},\gamma ,\text{update\_u}=\text{True}\right)$

**10**  update baseline offsets: $\beta \leftarrow {\text{min}}_{t}{\left(Y-U_{\text{base}}V_{\text{base}}-U_{\text{stim}}V_{\text{stim}}\right)}_{t}$

**11**  *β* ← max(*β*, 0)

**12**  **for** *r = 1*,…,*R* **do**

**13**   Define residual matrix $\tilde{Y}=Y_{0:t_{2}}-U_{\text{base}}V_{\text{base}}-\sum_{j\neq r}U_{\text{stim},j}V_{\text{stim},j}$

**14**   Update component *r* of current-trial photocurrent estimate $U_{\text{stim},r},V_{\text{stim},r}=\text{NMU-Coordinate-Descent}\left(\tilde{X},U_{\text{stim},r},V_{\text{stim},r},\gamma ,\text{update\_u}=\text{True},t_{3}\right)$

**15**  **end**

**16** **end**

**17** **return** $U_{\text{base}},V_{\text{base}},U_{\text{stim}},V_{\text{stim}},\beta$

**Algorithm 3:** NMUDecreasing

**input**: Data matrix *Y*, initial column vector *u*, initial row vector *v*, update_V=True, decrement *γ*, decrement start *t*_3_

**1 if** *Initial column vector u is None or initial row vector v is None* **then**

**2**  Initialize *u* and *v* using the SVD of *X*

**3 end**

**4** Initialize auxiliary variables:

**5** Γ ← zeros_like(*X*)

**6** λ ← zeros_like(*v*)

**7** *q* ← *v*

**8** *R* ← max(0, *Y* − *uv*)

**9** *k* ← 0

**10** **while** *not converged* **do**

**11**  *M* ← *Y* − *R* + Γ/*ρ*

**12**  Compute the matrix of residuals *L* ← *Y* − *uv*

**13**  Update and normalize *u*:

**14**  *u* ← *M* ⋅ *v*^*T*^

**15**  *u* ← max(0, *u*)

**16**  *u* ← *u*/‖*u*‖_2_

**17**  **if** *update_V* **then**

**18**   Update *v*:

**19**   *v* ← (*u*^*T*^ ⋅ (Γ + *ρ* ⋅ (*Y* − *R*)) + λ+ *ρ* ⋅ *q*)/(*ρ* ⋅ (*u*^*T*^ ⋅ *u* + 1))

**20**   *v* ← max(0, *v*)

**21**   Update auxiliary variable *q*:

**22**   *q*[0: *t*_3_]←(*v* − λ/*ρ*)[0, 0: *t*_3_]

**23**   *q*[*t*_3_:] ← pava_decreasing(*v*[0, *t*_3_:] −λ/*ρ*, *γ*)

**24**   Update Lagrange multiplier:

**25**   λ ← λ + *ρ* ⋅ (*q* − *v*)

**26**  **end**

**27**  Update *R*:

**28**  *R* ← 1/(1 + *ρ*) ⋅ (*ρ* ⋅ *L* + Γ)

**29**  *R* ← max(0, *R*)

**30**  Update Γ:

**31**  Γ ← Γ + *ρ* ⋅ (*L* − *R*)

**32 end**

**33** **return** *u*, *v*

#### Batched photocurrent subtraction

As shown in Fig 2, we observed slight power-dependent changes in photocurrent kinetics. We therefore adopted a batching strategy when estimating photocurrents, which we observed to improve performance on real data. To begin, we sort all photocurrents by the energy in the signal during stimulation. We then run the PhoRC algorithm (Alg 1) on batches of sorted traces. For all experiments shown, we used a batch size of 200 traces.

#### Setting PhoRC hyperparameters

Two main hyperparameters determine how aggressive PhoRC is when subtracting photocurrents. These are the photocurrent integration window (see Validating PhoRC using simulated mapping experiments) and the rank of the approximation (see The PhoRC algorithm).

Recall that PhoRC proceeds in two steps, first using a short window of data (the integration window) to learn the photocurrent weights *U* and subsequently estimating the waveform *V* with *U* held fixed. Longer integration windows result in more complete photocurrent removal, at the expense of occasionally subtracting low-latency PSCs. In our results, we used a 5 ms integration window on all grid datasets, and a 3 ms integration window on all targeted datasets, matching the respective pulse durations used in those experiments. Practitioners using stimulus durations longer than 5 ms may find it beneficial to reduce the integration window to end around 5 ms, to avoid erroneously subtracting PSCs.

Including more components in the low-rank approximation also makes subtraction more complete, at the expense of sometimes partially subtracting PSCs. We found that when photocurrents were vastly larger than PSCs, a rank-two approximation worked best, possibly because there was more variability in the photocurrent waveform in those cases. In cases where photocurrents were around the same magnitude as PSCs, a rank-one approximation worked well. We used rank-two approximations for all of the grid experiments, with the exception of cell 3, for which we found a rank-one approximation to work better (see S3 Fig). We used rank-one approximations for the targeted experiments.

### Connectivity inference and estimation of putative connection waveforms

To assess the impact of photocurrents on connectivity, we relied on the CAVIaR pipeline from [7]. This pipeline comprises two stages: neural waveform demixing (NWD), and connectivity inference. The NWD stage uses a neural network to denoise data and correct for confounded baselines when using short ISIs. This stage also subtracts any PSCs which are not initiated within a window characteristic of monosynaptic transmission, typically 3–12 ms. The connectivity inference stage uses a statistical model to infer which trials were successful in eliciting an upstream spike, and subsequently to learn an effective synaptic weight for each stimulation target. In all results, we used both stages of the pipeline when inferring connectivity (e.g Figs 3 and 7). In the presenence of photocurrent artifacts, we found that combining PhoRC with NWD yielded better results than applying either technique independently.

As described in [7], the connectivity inference stage of CAVIaR allows for the computation of putative connection waveforms as follows. During connectivity inference, CAVIaR infers a matrix Λ, in which Λ_*ij*_ represents the posterior probability that cell *i* spiked on trial *j*. Using this presynaptic spike matrix **Λ**, we can obtain accurate PSC waveforms $r_{n}\in R^{T}$ using ridge regression. Collecting the waveforms in the rows of a matrix **R**, we obtain an estimate for **R** by solving the *L*_2_ problem

$$\begin{array}{c} \hat{R}=\underset{R\geq 0}{\text{arg min}}\{∥C-\Lambda^{⊤}{R∥}_{F}+\lambda {∥R∥}_{F}\}. \end{array}$$ (18)

In all results showing connection waveforms, we computed this regression using only trials at the highest laser power.

### Selecting representative traces

In order to demonstrate PhoRC’s performance, we found it useful to examine the model’s behavior on a small subset of traces collected during the experiment. In most cases, we extracted traces from two categories: those with the largest putative synaptic current components, and those with the the largest putative photocurrent components.

To extract traces with large synaptic currents, we first ran PhoRC to remove photocurrents, and then applied NWD to denoise and correct baselines. NWD is trained to isolate PSCs which onset in an “admissible window” around stimulation onset, and thus it tends to reduce synaptic currents caused by spontaneous activity [7]. We thus treated traces with the largest NWD outputs as being likely to contain evoked synaptic currents.

To extract traces with large putative photocurrents, we simply used the magnitude of the PhoRC estimate, restricted to the time during stimulation. Thus, wherever we plotted representative traces (e.g Figs 3 and 7, and S1–S6 Figs), we included 10–15 traces with the largest NWD outputs, and 10–15 traces with the largest PhoRC outputs.

### Validation with simulated connnectivity mapping experiments

For the simulated results in Fig 5, we simulated a continuous mapping experiment with a population of 100 presynaptic neurons, assigned at random to be connected to the postsynaptic cell with probability 0.1. Using the connectivity mapping simulator from [7], we obtain simulated PSCs and electrical noise for each trial. In the interest of brevity, we refer the reader to [7] for a detailed description of these connectivity mapping simulations. We note that these simulations assign a canonical PSC waveform to each connected cell, and that this waveform has power-dependent latency as we observed in the real data (see e.g Fig 1).

A fraction of cells were chosen with probability *p* (the “photocurrent fraction”) to evoke direct photocurrent in the postsynaptic cell. Concretely, let *q*_*n*_ = 1 if cell *n* evokes photocurrent; then

$$\begin{array}{c} q_{n}∼\text{Bernoulli}\left(p\right). \end{array}$$ (19)

Conditional on *q*_*n*_ = 1, the maximal mean evoked photocurrent for presynaptic cell *n* was drawn from a gamma distribution:

$$\begin{array}{c} \mu_{n}∼\text{Gamma}\left(k_{\text{photo}},\theta_{\text{photo}}\right). \end{array}$$ (20)

Finally, we draw the photocurrent amplitude contributed from presynaptic target *n* on trial *k* as

$$\begin{array}{c} A_{k}∼\text{Normal}\left(\frac{I_{k}}{I_{\text{max}}}\mu_{n},\sigma_{A}^{2}\right), \end{array}$$ (21)

where *I*_max_ represents the maximum power used during the experiment, and *I*_*k*_ is the laser power on trial *k*. We can occasionally sample values for *A*_*k*_ which are below zero, so we simply take these to be zero. Thus *μ*_*n*_ represents the average evoked photocurrent amplitude when stimulating at the highest laser power. This model introduces variability, controlled by $\sigma_{A}^{2}$, when stimulating the same presynaptic target, as we observe in the real data. The computed amplitude *A*_*k*_ is used to scale the photocurrent waveform, as we describe below.

In order to account for prior and next-trial effects, we simulated a continuous intracellular voltage recording, which was then broken down into overlapping 45ms snippets. This captures scenarios in which photocurrents and PSCs from the prior trial may not have decayed to zero before the next stimulus. Simulated photocurrents used a variant of the double two-state opsin model described by [16], represented by the following set of equations:

$$\begin{array}{c} O_{\text{on}}=\left(\Theta \left(t-t_{\text{on}}\right)-\Theta \left(t-t_{\text{off}}\right)\right)\left(O_{\infty }-\left(O_{\infty }-O_{0}\right)e^{-\left(t-t_{\text{on}}\right)/\tau_{o}}\right), \end{array}$$ (22)

$$\begin{array}{c} O_{\text{off}}=\Theta \left(t-t_{\text{off}}\right)O_{\text{on}}\left[t_{\text{off}}\right]e^{-\left(t-t_{\text{off}}\right)/\tau_{o}}, \end{array}$$ (23)

$$\begin{array}{c} R_{\text{on}}=\left(\Theta \left(t-t_{\text{on}}\right)-\theta \left(t-t_{\text{off}}\right)\right)\left(R_{\infty }-\left(R_{\infty }-R_{0}\right)e^{-\left(t-t_{\text{on}}\right)/\tau_{r}}\right), \end{array}$$ (24)

$$\begin{array}{c} R_{\text{off}}=\Theta \left(t-t_{\text{off}}\right)\left(1-\left(1-R_{\text{on}}\left[t_{\text{off}}\right]\right)e^{-\left(t-t_{\text{off}}\right)/\tau_{r}}\right), \end{array}$$ (25)

$$\begin{array}{c} I_{\text{photo}}=\left(O_{\text{on}}+O_{\text{off}}\right)\left(R_{\text{on}}+R_{\text{off}}\right), \end{array}$$ (26)

where *Θ*(*t*) is the heaviside step function, and *I*_photo_ is the induced photocurrent. The parameters governing the shape of the photocurrent waveform, *R*_inf_, *O*_inf_, *τ*_*o*_, *τ*_*r*_, were selected uniformly at random from a range of values to match variability in photocurrent kinetics in the real data (see Fig 2). For each simulated experiment, we re-sampled values of these parameters, giving a simulated postsynaptic cell its own photocurrent waveform. At each trial, we add scaled photocurrents *A*_*nk*_*I*_photo_ to simulated PSCs and noise. In our simulations, we use a 5ms pulse duration, and include 5 ms of context from the prior trial so that *t*_on_ = 5 ms and *t*_off_ = 10 ms.

### Validation with hybrid data

For the hybrid data validation presented in Fig 6, we began with opsin-negative grid mapping datasets collected using the Ai203 mouse line [28] and added synthetic photocurrents on top of recorded ground-truth traces. For these simulations, it was important that the photocurrent contamination had a spatial footprint, as we see in the real data. To accomplish this, we use a three dimensional Gaussian density to modulate the mean evoked photocurrent at each point in space. Let **x**_*k*_ be a point on a three-dimensional grid stimulated during trial *k*, let *I*_*k*_ be the power delivered at trial *k*, and let **c** be the location of the postsynaptic cell. We define the mean photocurrent amplitude at a given location and laser power to be

$$\begin{array}{c} \mu_{k}=[\text{exp}(-{\left(x_{k}-c\right)}^{⊤}\Sigma^{-1}\left(x_{k}-c\right))][\phi_{0}\sigma \left(I_{k}-\phi_{1}\right)]. \end{array}$$ (27)

Here, *μ*_*k*_ is a mean used to sample the photocurrent amplitude, Σ is a diagonal matrix determining the spatial extent of the photocurrent response. *ϕ*_1_ and *ϕ*_0_ are parameters governing how *μ*_*k*_ changes with laser power, and *σ* is the sigmoid function. Thus, the first bracketed term scales *μ*_*k*_ with distance from the postsynaptic cell, and the second bracketed term scales *μ*_*k*_ with laser power. To introduce random variability when stimulating the same target location, we sample *A*_*k*_, the photocurrent amplitude on trial *k*, as

$$\begin{array}{c} A_{k}∼\text{Normal}\left(\mu_{k},\sigma_{A}^{2}\right). \end{array}$$ (28)

Using simulated photocurrents from Eq 26, we added these scaled photocurrents to the ground-truth grid maps. We then ran PhoRC and computed mean-squared error (MSE) by comparing the recovered PSC responses to the ground-truth data.

## Supporting information

S1 Fig Cell 1 subtraction detail with comparison between single-target and compressed-sensing based mapping. Same dataset as shown in Figs 1, 3 and 7. **a**, Single-target mapping. Raw, estimated, and subtracted maps are shown for five planes (rows) and three powers (columns). The rightmost column shows the result of applying CAVIaR to estimate synaptic weights, which effectively merges data across laser powers. Patched cell is on plane 50 *μ*m. **b**, Raw, estimated photocurrents, and subtracted traces for highest laser power. Since the photocurrents and estimates are so much larger than synaptic currents, the rightmost column shows the subtracted traces rescaled for legibility. Traces were selected by finding the 15 largest estimated synaptic responses, and the 15 largest estimated photocurrents. **c**, Same as **a** for ensemble mapping with the same postsynaptic cell. For multispot data, raw and subtracted maps are created by averaging all ensembles in which a given voxel was stimulated, thus we expect these maps to appear “noisier” than in the single-target case. **d**, Same as **b** for ensemble mapping of the same postynaptic cell.
(PDF)

S2 Fig Cell 2 subtraction detail. **a** Full grid mapping dataset showing subtraction performance. Raw, estimated, and subtracted maps are shown for five planes (rows) and three powers (columns). For legibility, color-scale is truncated to match the range of the subtracted data. The rightmost column shows the result of applying CAVIaR to estimate synaptic weights, which effectively merges data across laser powers. Patched cell is on plane 50 *μ*m. **b** Raw, estimated photocurrents, and subtracted traces for three powers. Traces were selected by finding the 15 largest estimated synaptic responses, and the 15 largest estimated photocurrents. As seen in the bottom row, PhoRC occasionally subtracts traces with corrupted baselines.
(PDF)

S3 Fig Cell 3 subtraction detail. **a** Full grid mapping dataset showing subtraction performance. Raw, estimated, and subtracted maps are shown for three powers (columns). For legibility, color-scale is truncated to match the range of the subtracted data. The rightmost column shows the result of applying CAVIaR to estimate synaptic weights, which effectively merges data across laser powers. Patched cell is on plane 50 *μ*m. **b** Raw, estimated photocurrents, and subtracted traces for three powers. Traces were selected by finding the 15 largest estimated synaptic responses, and the 15 largest estimated photocurrents. Note that in some cases PhoRC mistakenly captures very low-latency PSCs. This is expected behavior, since these PSCs occur concurrently with stimulation onset (and are therefore likely indicative of spontaneous activity rather than a monosynaptic connection).
(PDF)

S4 Fig Cell 4 subtraction detail. **a** Full grid mapping dataset showing subtraction performance. Raw, estimated, and subtracted maps are shown for five planes (rows) and three powers (columns). For legibility, color-scale is truncated to match the range of the subtracted data. The rightmost column shows the result of applying CAVIaR to estimate synaptic weights, which effectively merges data across laser powers. Patched cell is on plane 50 *μ*m. **b** Raw, estimated photocurrents, and subtracted traces for three powers. Traces were selected by finding the 15 largest estimated synaptic responses, and the 15 largest estimated photocurrents.
(PDF)

S5 Fig Cell 5 subtraction detail with synaptic transmission block. **a** Full grid mapping dataset during the control block. Raw, estimated, and subtracted maps are shown for five planes (rows) and three powers (columns). For legibility, color-scale is truncated to match the range of the subtracted data. Patched cell is on plane 50 *μ*m. **b** Raw, estimated photocurrents, and subtracted traces for the highest laser power. Traces were selected by taking the responses with the largest estimated photocurrent component. **c**, Same as **a** but during the NBQX block. **d**, Same as **b** but during the NBQX block. Traces were selected by taking the responses with the largest estimated photocurrent component.
(PDF)

S6 Fig Photocurrent subtraction for pyramidal to PV mapping. This dataset represents a putative PV patch, recognizable by the very sharply decaying PSCs. **a** Full grid mapping dataset showing subtraction performance. Raw, estimated, and subtracted maps are shown for five planes (rows) and three powers (columns). For legibility, color-scale is truncated to match the range of the subtracted data. The rightmost column shows the result of applying CAVIaR to estimate synaptic weights, which effectively merges data across laser powers. Patched cell is on plane 50 *μ*m. **b** Raw, estimated photocurrents, and subtracted traces for three powers. Traces were selected by finding the 15 largest estimated synaptic responses, and the 15 largest estimated photocurrents. As seen in the middle row, PhoRC occasionally subtracts PSCs which occur around the time of stim onset. This is to be expected, and their very low latencies suggest that these PSCs are likely due to spontaneous activity.
(PDF)

S7 Fig Cell 1 subtraction detail showing effects of batch size. **a** Largest traces (sorted by total signal amplitude) from each power in a single grid experiment. Twenty traces are displayed for each laser power. **b** Traces from **a** for each power were averaged, then normalized to lie between zero and one. We observed slight differences in photocurrent onset across powers. **c** To ensure that these slight kinetic differences did not impact photocurrent estimates, we sorted traces by signal magnitude (sum of the signal during the stim period) and ran PhoRC on batches of traces with similar magnitudes. Raw, photocurrent estimates, and subtracted traces are shown across a variety of batch sizes. Results are shown with rank = 2.
(PDF)

S8 Fig Effects of varying batch size on E to PV mapping. Application of PhoRC to a putative E to PV mapping experiment. Columns show raw traces, photocurrent estimates, and subtracted traces respectively. Rows show varying batch sizes. Twenty largest responses at each laser power (sorted by signal sum during stim) are presented. At very small batch sizes (10–20), PhoRC can erroneously subtract PSCs. However, performance for batch sizes with 200 or greater traces appears similar.
(PDF)

S9 Fig Effects of varying batch size with ensemble stimualtion. Same as S8 Fig, but for an ensemble stimulation experiment. Columns show raw traces, photocurrent estimates, and subtracted traces respectively. Rows show varying batch sizes. Twenty largest responses at each laser power (sorted by signal sum during stim) are presented. Performance at all batch sizes appears similar in this case.
(PDF)

S10 Fig Accounting for prior and subsequent trial effects. The integration window begins at the time of laser onset (*t*_1_) and ends at a user-defined time (*t*_2_), typically chosen to be 3–5ms after laser onset. To avoid including photocurrents or synaptic currents from the subsequent trial, we enforce an exponential decay constraint beginning at time *t*_3_.
(PDF)
