# Supplementary figures and images for: Removing direct photocurrent artifacts in optogenetic connectivity mapping data via constrained matrix factorization
Source: PLoS Comput Biol. 2024 May 6;20(5):e1012053. doi: 10.1371/journal.pcbi.1012053 (PMC11098512; doi:10.1371/journal.pcbi.1012053)

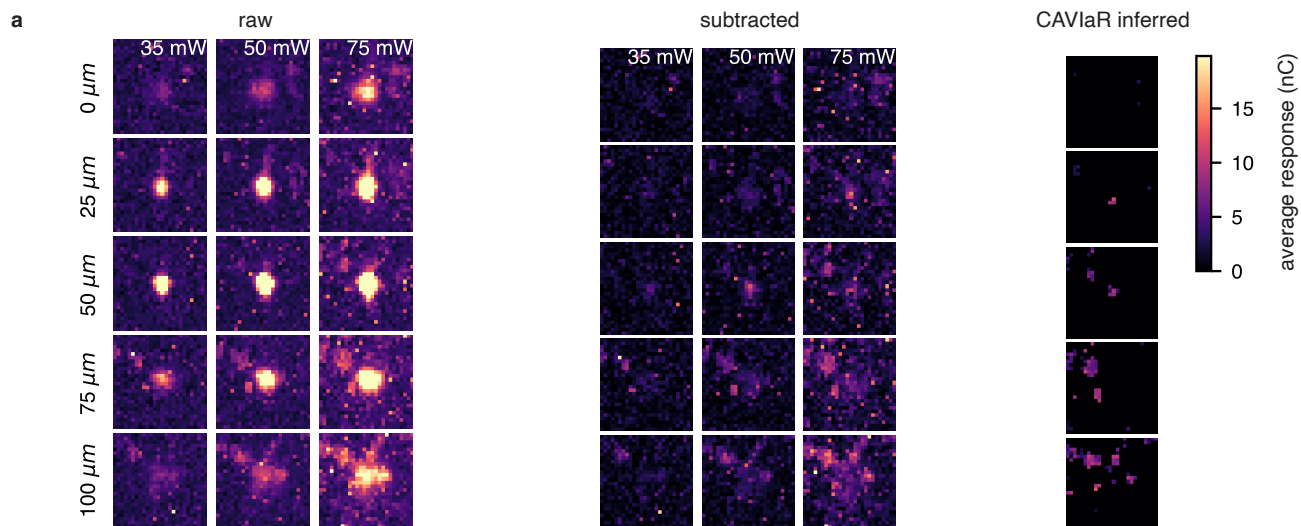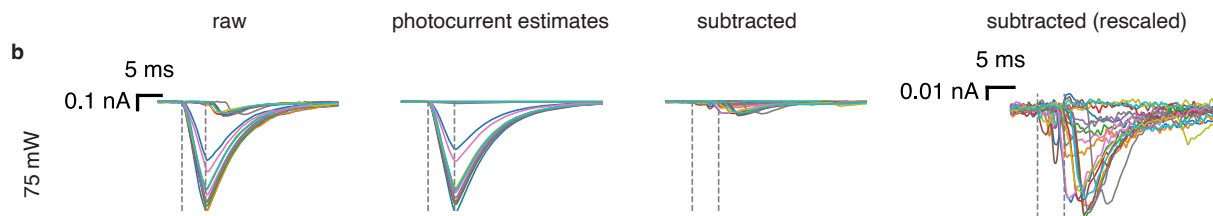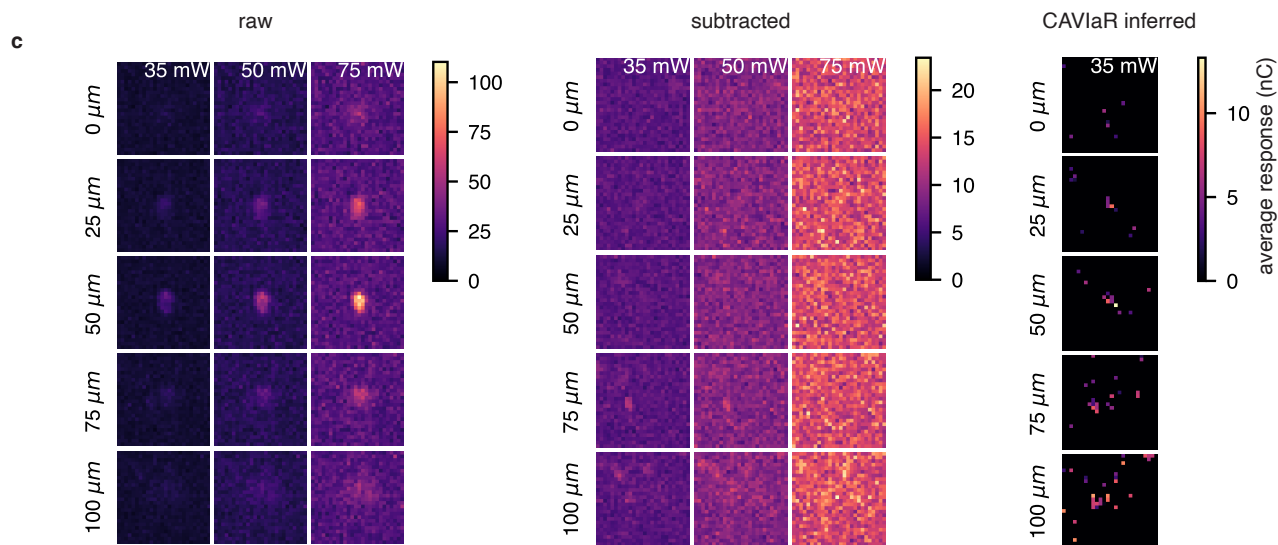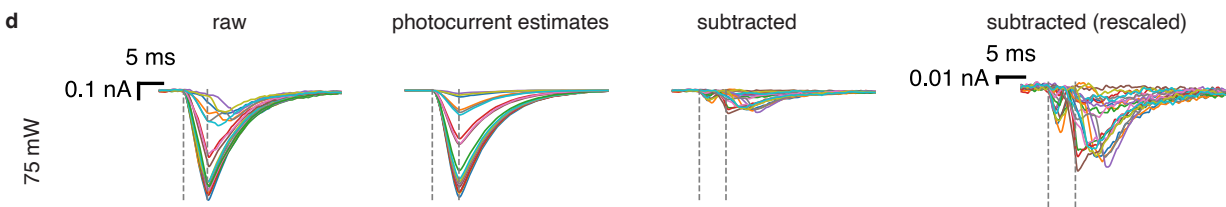

Supplement: S1 Fig — Same dataset as shown in Figs 1, 3 and 7. a, Single-target mapping. Raw, estimated, and subtracted maps are shown for five planes (rows) and three powers (columns). The rightmost column shows the result of applying CAVIaR to estimate synaptic weights, which effectively merges data across laser powers. Patched cell is on plane 50 μm. b, Raw, estimated photocurrents, and subtracted traces for highest laser power. Since the photocurrents and estimates are so much larger than synaptic currents, the rightmost column shows the subtracted traces rescaled for legibility. Traces were selected by finding the 15 largest estimated synaptic responses, and the 15 largest estimated photocurrents. c, Same as a for ensemble mapping with the same postsynaptic cell. For multispot data, raw and subtracted maps are created by averaging all ensembles in which a given voxel was stimulated, thus we expect these maps to appear “noisier” than in the single-target case. d, Same as b for ensemble mapping of the same postynaptic cell. (PDF) [file pcbi.1012053.s001.pdf]

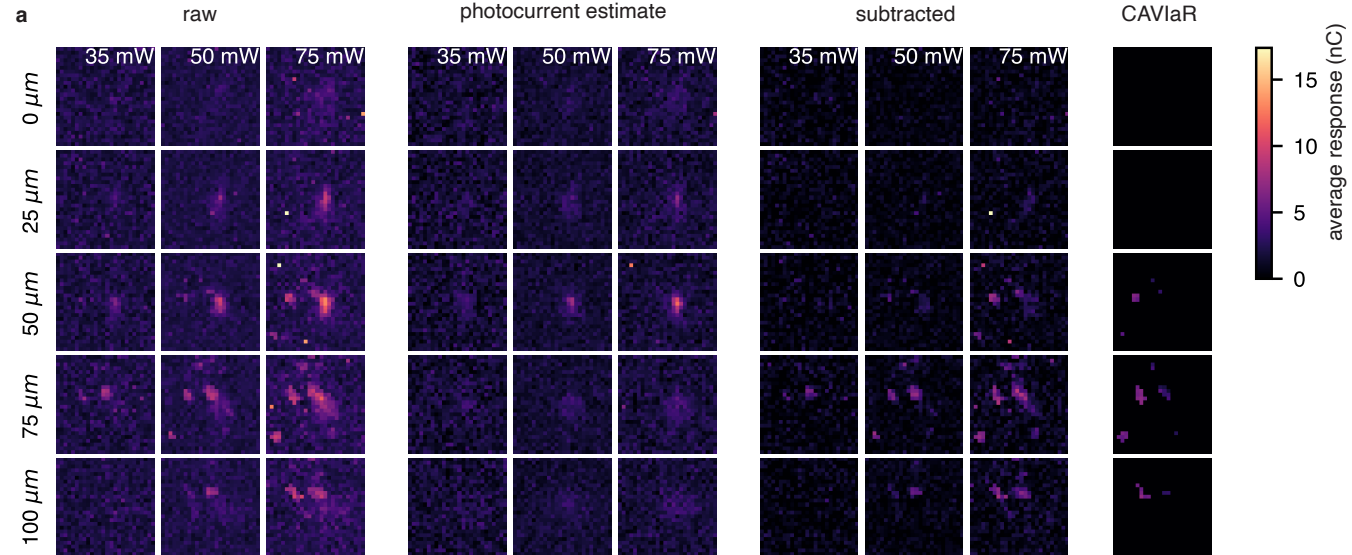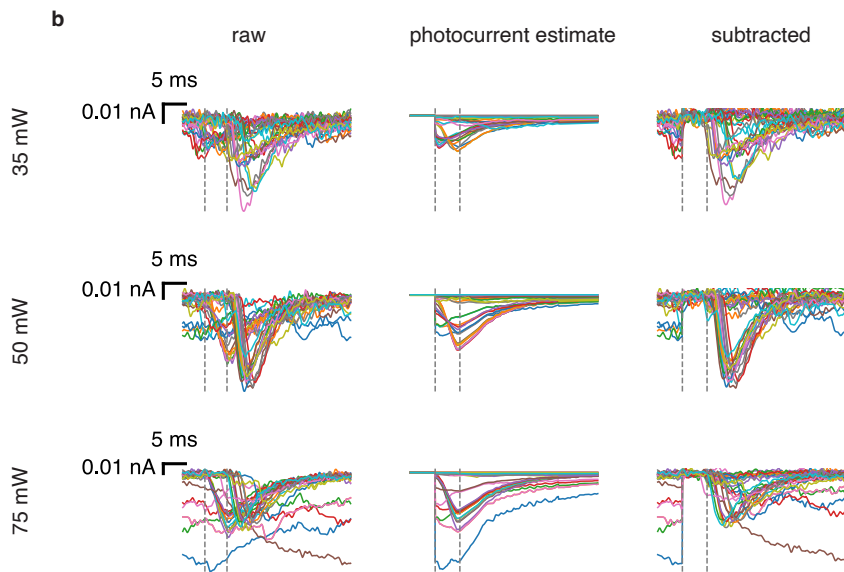

Supplement: S2 Fig — a Full grid mapping dataset showing subtraction performance. Raw, estimated, and subtracted maps are shown for five planes (rows) and three powers (columns). For legibility, color-scale is truncated to match the range of the subtracted data. The rightmost column shows the result of applying CAVIaR to estimate synaptic weights, which effectively merges data across laser powers. Patched cell is on plane 50 μm. b Raw, estimated photocurrents, and subtracted traces for three powers. Traces were selected by finding the 15 largest estimated synaptic responses, and the 15 largest estimated photocurrents. As seen in the bottom row, PhoRC occasionally subtracts traces with corrupted baselines. (PDF) [file pcbi.1012053.s002.pdf]

**a**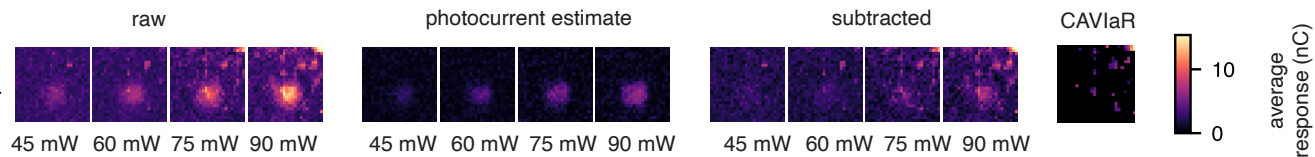**b**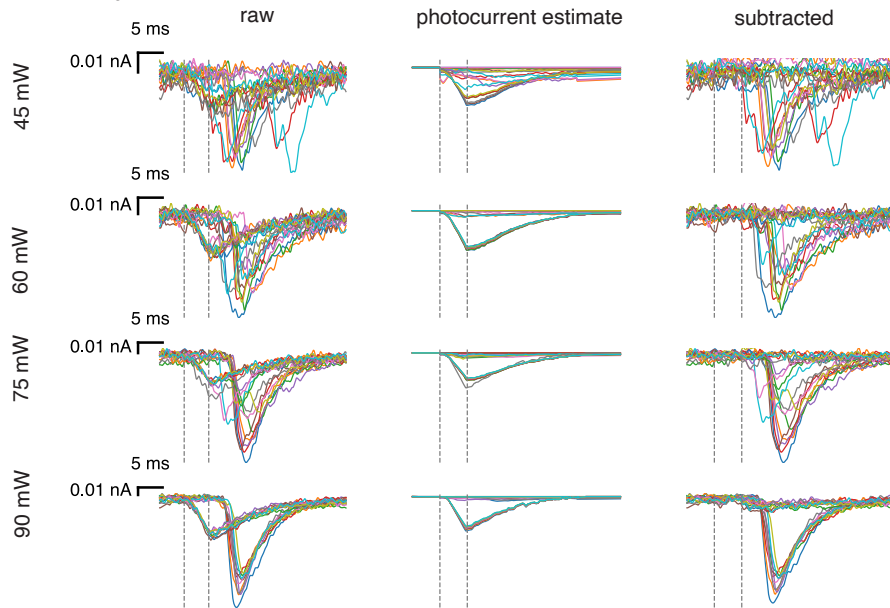

Supplement: S3 Fig — a Full grid mapping dataset showing subtraction performance. Raw, estimated, and subtracted maps are shown for three powers (columns). For legibility, color-scale is truncated to match the range of the subtracted data. The rightmost column shows the result of applying CAVIaR to estimate synaptic weights, which effectively merges data across laser powers. Patched cell is on plane 50 μm. b Raw, estimated photocurrents, and subtracted traces for three powers. Traces were selected by finding the 15 largest estimated synaptic responses, and the 15 largest estimated photocurrents. Note that in some cases PhoRC mistakenly captures very low-latency PSCs. This is expected behavior, since these PSCs occur concurrently with stimulation onset (and are therefore likely indicative of spontaneous activity rather than a monosynaptic connection). (PDF) [file pcbi.1012053.s003.pdf]

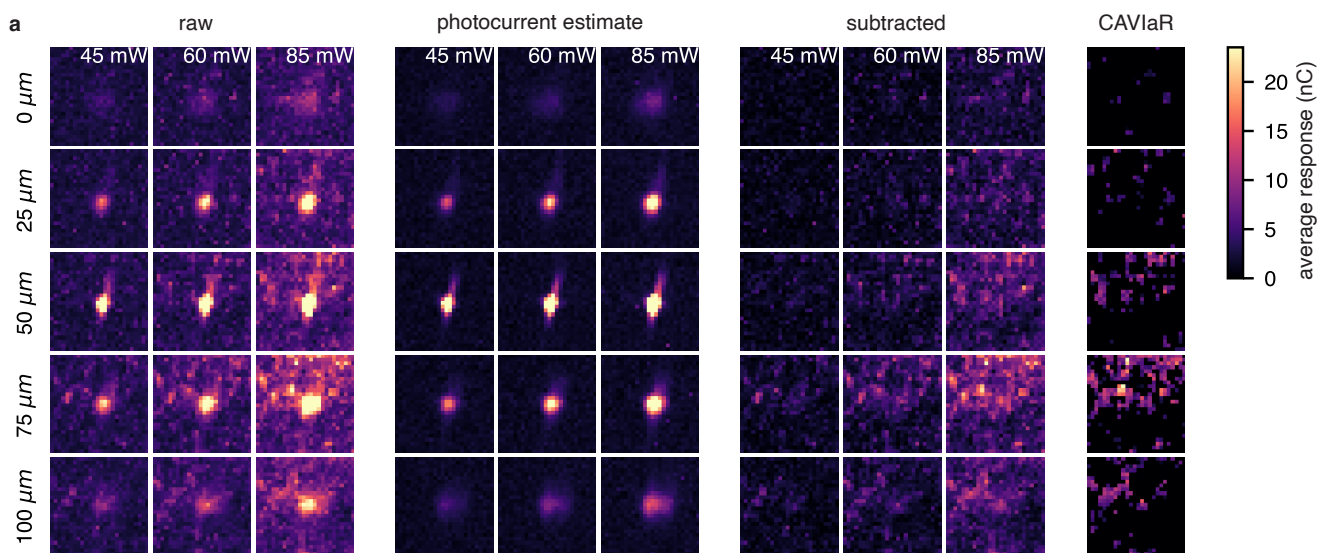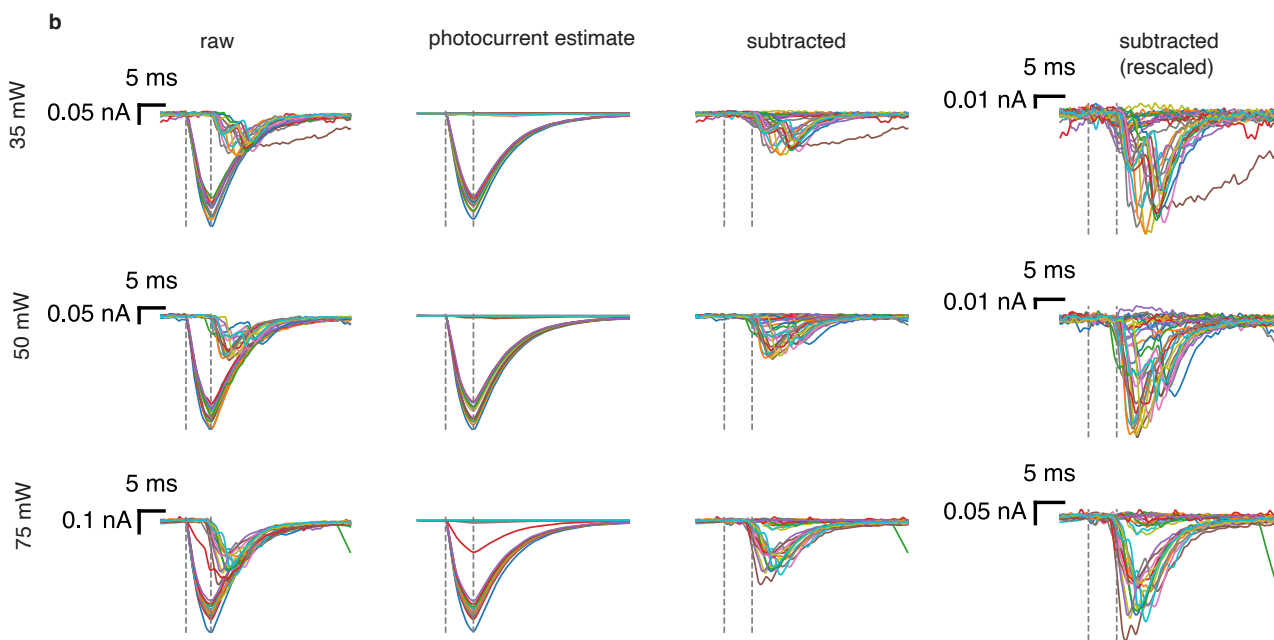

Supplement: S4 Fig — a Full grid mapping dataset showing subtraction performance. Raw, estimated, and subtracted maps are shown for five planes (rows) and three powers (columns). For legibility, color-scale is truncated to match the range of the subtracted data. The rightmost column shows the result of applying CAVIaR to estimate synaptic weights, which effectively merges data across laser powers. Patched cell is on plane 50 μm. b Raw, estimated photocurrents, and subtracted traces for three powers. Traces were selected by finding the 15 largest estimated synaptic responses, and the 15 largest estimated photocurrents. (PDF) [file pcbi.1012053.s004.pdf]

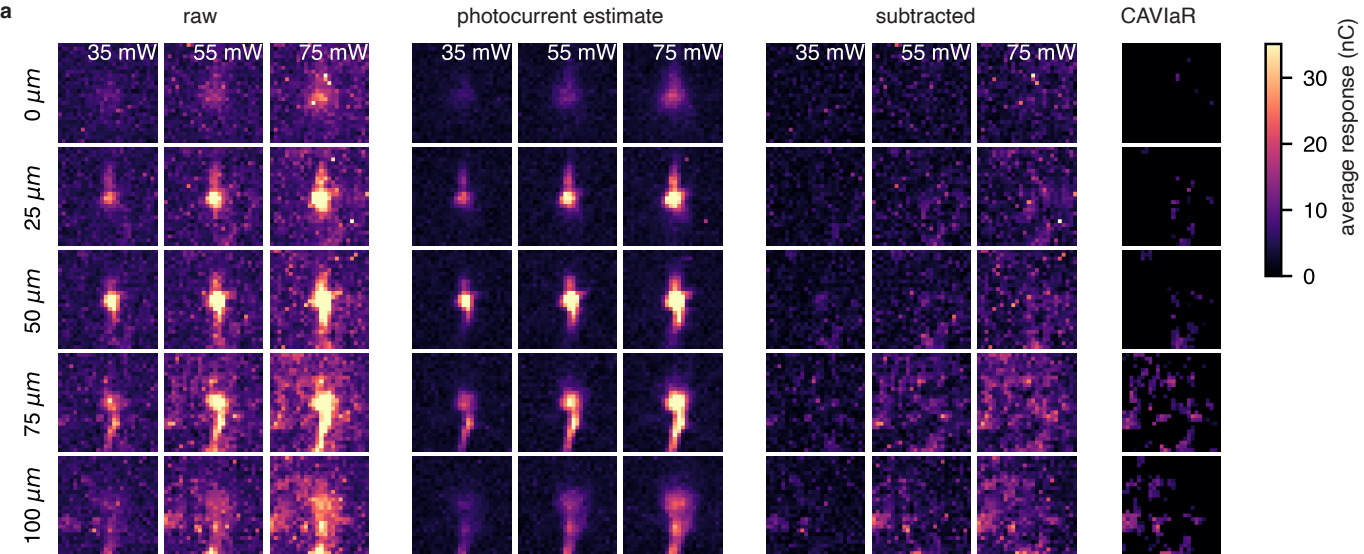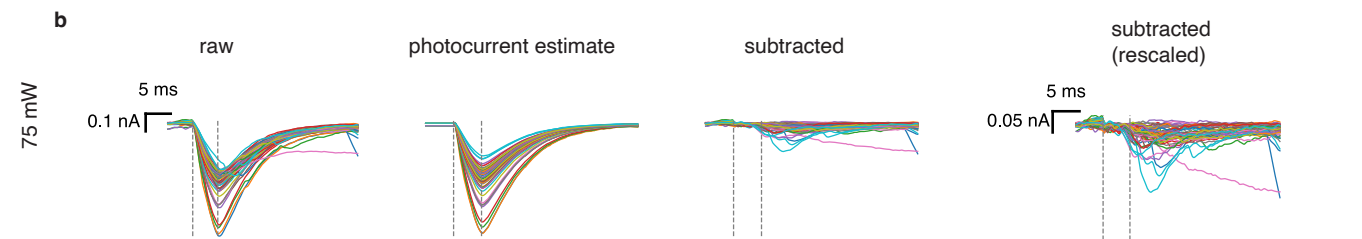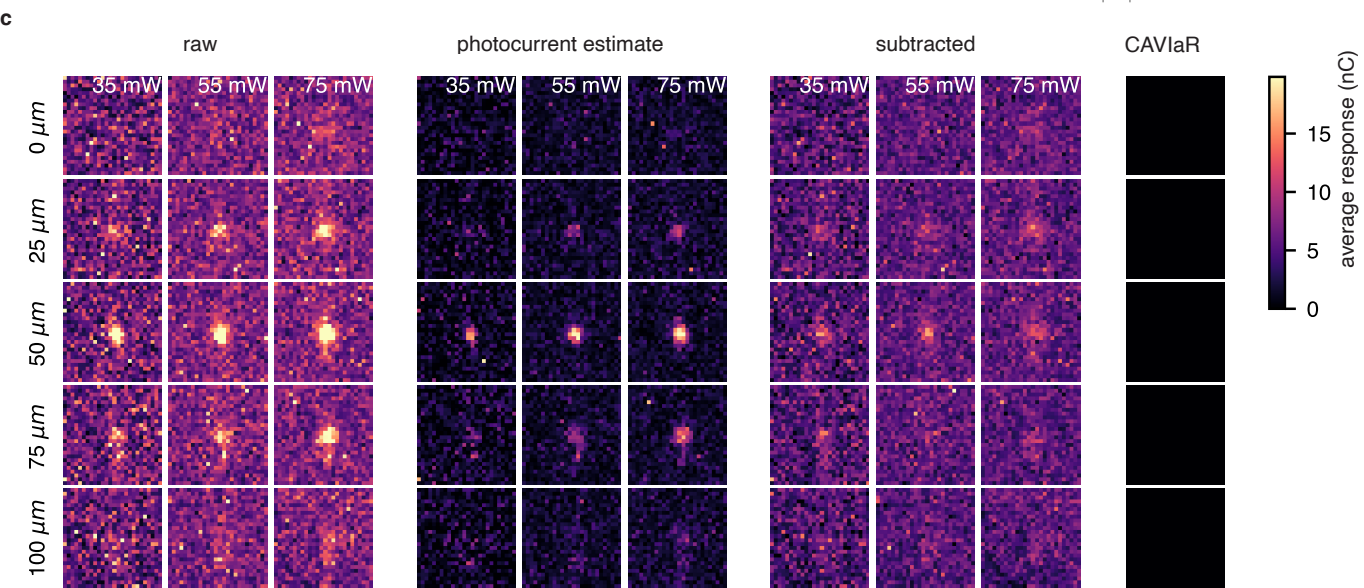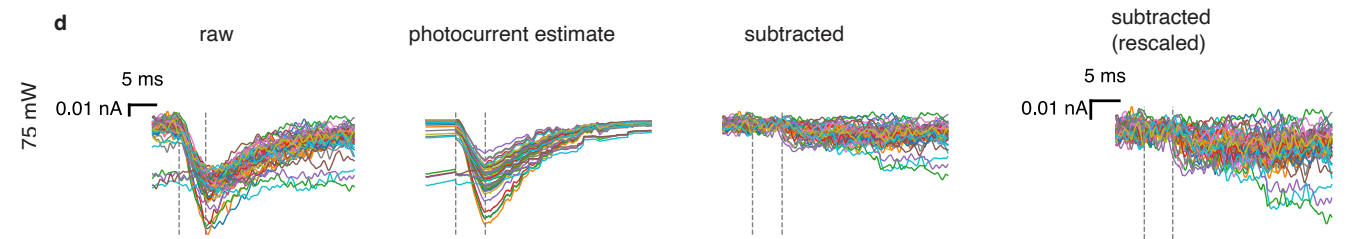

Supplement: S5 Fig — a Full grid mapping dataset during the control block. Raw, estimated, and subtracted maps are shown for five planes (rows) and three powers (columns). For legibility, color-scale is truncated to match the range of the subtracted data. Patched cell is on plane 50 μm. b Raw, estimated photocurrents, and subtracted traces for the highest laser power. Traces were selected by taking the responses with the largest estimated photocurrent component. c, Same as a but during the NBQX block. d, Same as b but during the NBQX block. Traces were selected by taking the responses with the largest estimated photocurrent component. (PDF) [file pcbi.1012053.s005.pdf]

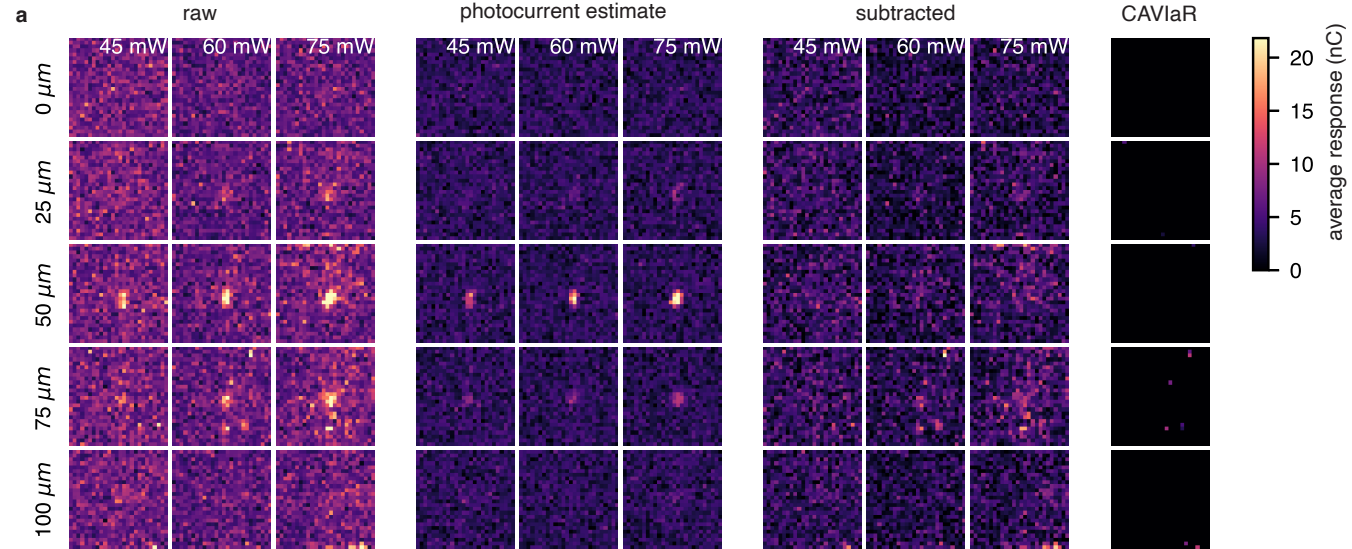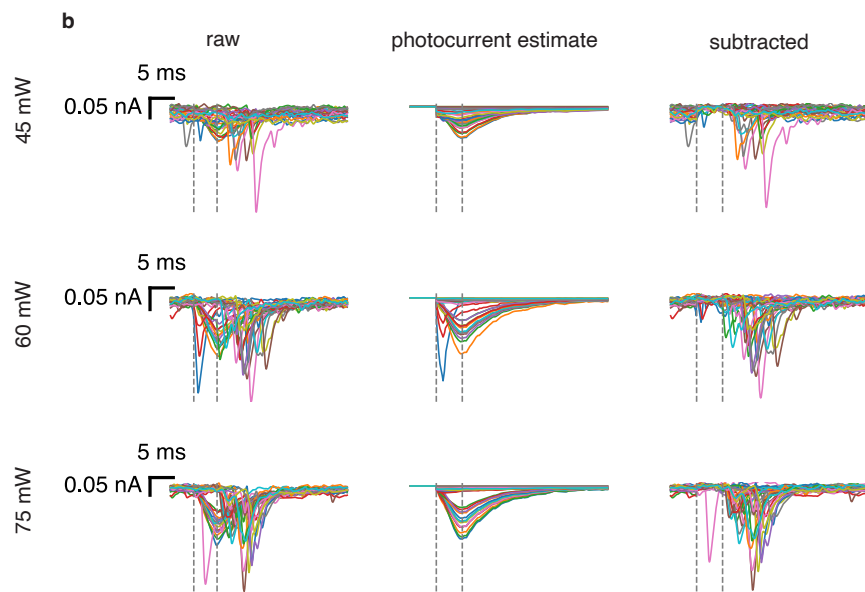

Supplement: S6 Fig — This dataset represents a putative PV patch, recognizable by the very sharply decaying PSCs. a Full grid mapping dataset showing subtraction performance. Raw, estimated, and subtracted maps are shown for five planes (rows) and three powers (columns). For legibility, color-scale is truncated to match the range of the subtracted data. The rightmost column shows the result of applying CAVIaR to estimate synaptic weights, which effectively merges data across laser powers. Patched cell is on plane 50 μm. b Raw, estimated photocurrents, and subtracted traces for three powers. Traces were selected by finding the 15 largest estimated synaptic responses, and the 15 largest estimated photocurrents. As seen in the middle row, PhoRC occasionally subtracts PSCs which occur around the time of stim onset. This is to be expected, and their very low latencies suggest that these PSCs are likely due to spontaneous activity. (PDF) [file pcbi.1012053.s006.pdf]

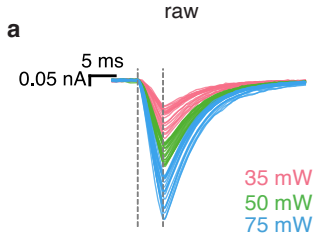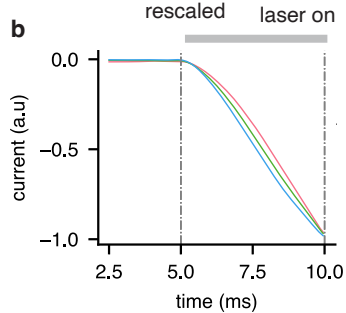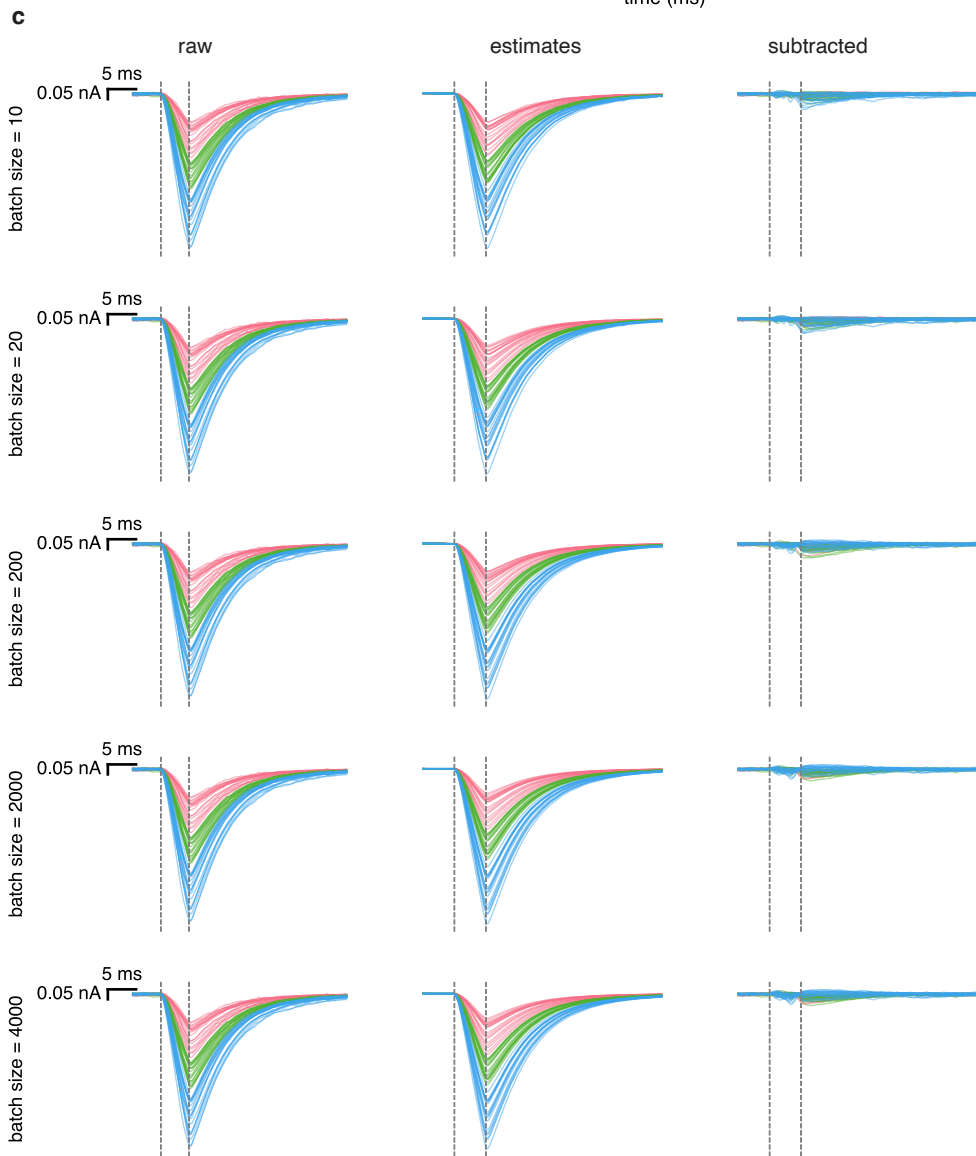

Supplement: S7 Fig — a Largest traces (sorted by total signal amplitude) from each power in a single grid experiment. Twenty traces are displayed for each laser power. b Traces from a for each power were averaged, then normalized to lie between zero and one. We observed slight differences in photocurrent onset across powers. c To ensure that these slight kinetic differences did not impact photocurrent estimates, we sorted traces by signal magnitude (sum of the signal during the stim period) and ran PhoRC on batches of traces with similar magnitudes. Raw, photocurrent estimates, and subtracted traces are shown across a variety of batch sizes. Results are shown with rank = 2. (PDF) [file pcbi.1012053.s007.pdf]

raw

estimates

subtracted

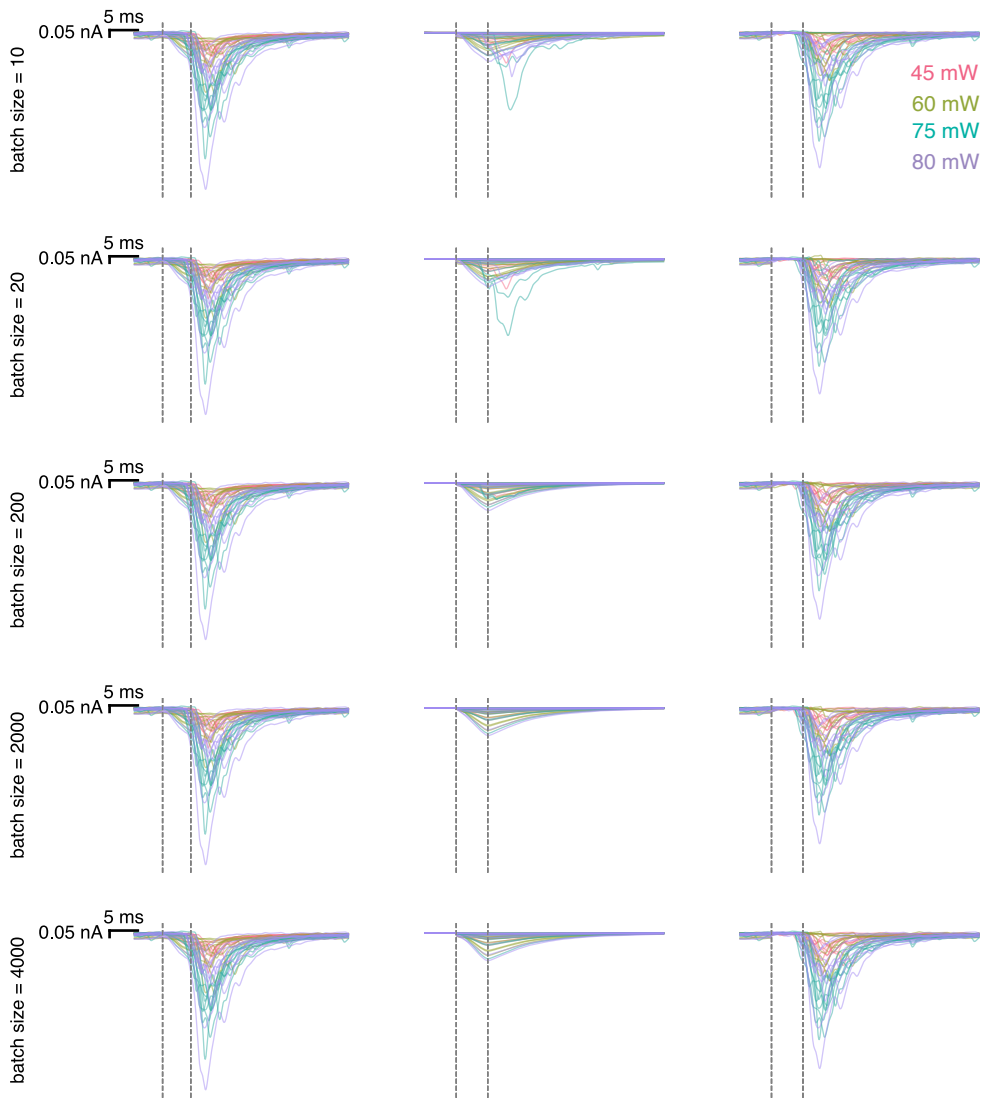

Supplement: S8 Fig — Application of PhoRC to a putative E to PV mapping experiment. Columns show raw traces, photocurrent estimates, and subtracted traces respectively. Rows show varying batch sizes. Twenty largest responses at each laser power (sorted by signal sum during stim) are presented. At very small batch sizes (10–20), PhoRC can erroneously subtract PSCs. However, performance for batch sizes with 200 or greater traces appears similar. (PDF) [file pcbi.1012053.s008.pdf]

raw

estimates

subtracted

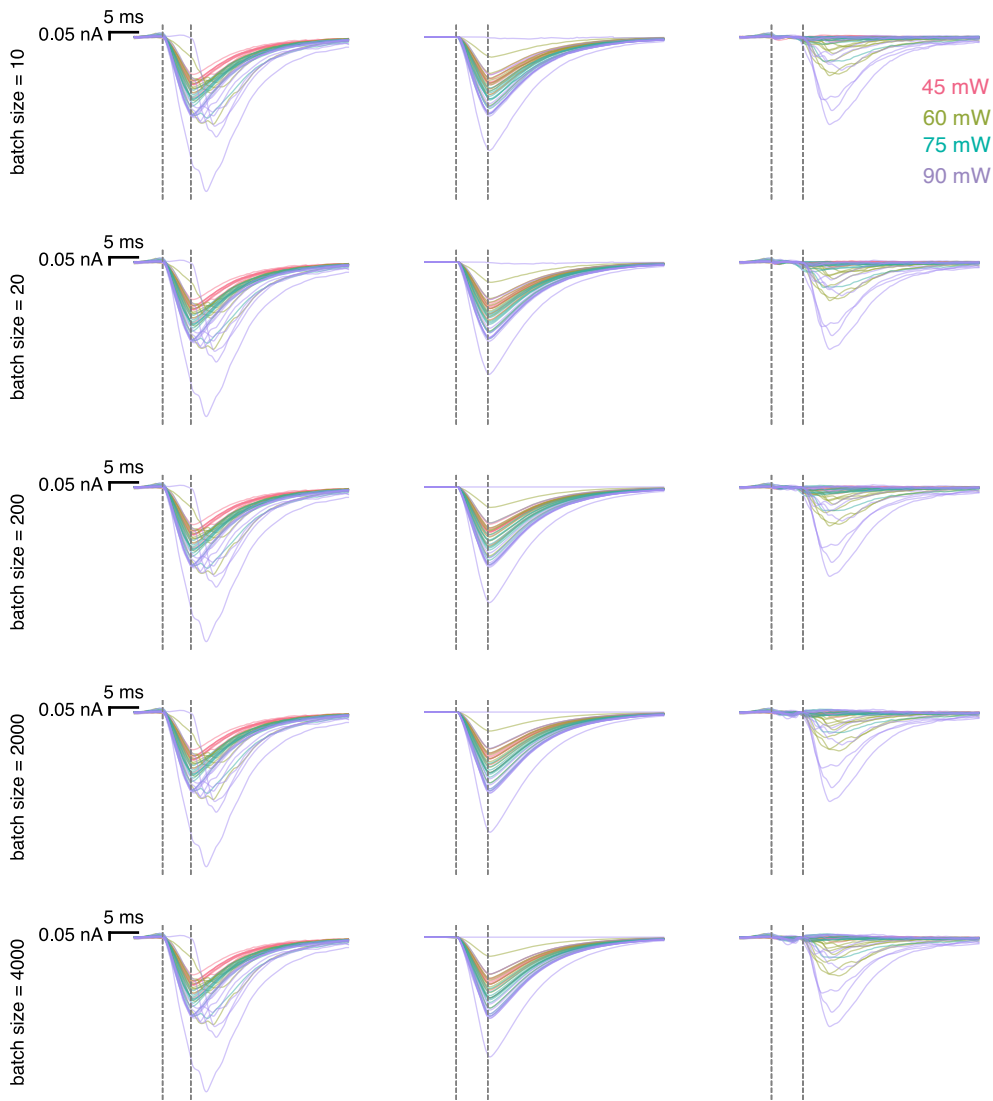

Supplement: S9 Fig — Same as S8 Fig, but for an ensemble stimulation experiment. Columns show raw traces, photocurrent estimates, and subtracted traces respectively. Rows show varying batch sizes. Twenty largest responses at each laser power (sorted by signal sum during stim) are presented. Performance at all batch sizes appears similar in this case. (PDF) [file pcbi.1012053.s009.pdf]

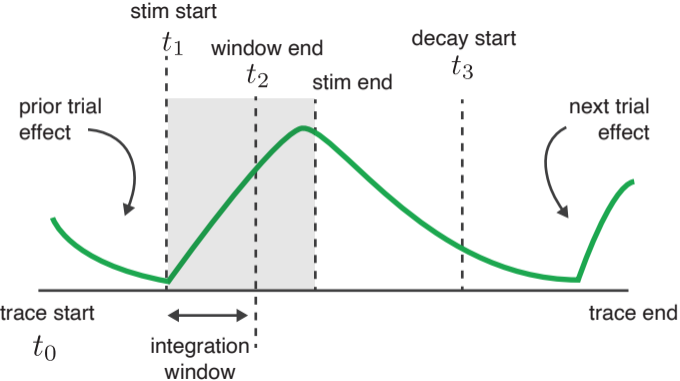

Supplement: S10 Fig — The integration window begins at the time of laser onset (t1) and ends at a user-defined time (t2), typically chosen to be 3–5ms after laser onset. To avoid including photocurrents or synaptic currents from the subsequent trial, we enforce an exponential decay constraint beginning at time t3. (PDF) [file pcbi.1012053.s010.pdf]
